# A novel broad-spectrum bacteriophage cocktail against methicillin-resistant *Staphylococcus aureus*: Isolation, characterization, and therapeutic potential in a mastitis mouse model

**DOI:** 10.1371/journal.pone.0316157

**Published:** 2025-01-15

**Authors:** Maryam Banar, Haniyeh Kamyab, Narges Torkashvand, Taghi Zahraei Salehi, Zargham Sepehrizadeh, Ahmad Reza Shahverdi, Mohammad Reza Pourmand, Mohammad Hossein Yazdi

**Affiliations:** 1 Department of Pathobiology, School of Public Health, Tehran University of Medical Sciences, Tehran, Iran; 2 Department of Pharmaceutical Biotechnology and Biotechnology Research Center, Faculty of Pharmacy, Tehran University of Medical Sciences, Tehran, Iran; 3 Department of Microbiology, Faculty of Veterinary Medicine, University of Tehran, Tehran, Iran; 4 Recombinant Vaccine Research Center, Faculty of Pharmacy, Tehran University of Medical Sciences, Tehran, Iran; South China Agricultural University, CHINA

## Abstract

Bovine mastitis is a considerable challenge within the dairy industry, causing significant financial losses and threatening public health. The increased occurrence of methicillin-resistant *Staphylococcus aureus* (MRSA) has provoked difficulties in managing bovine mastitis. Bacteriophage therapy presents a novel treatment strategy to combat MRSA infections, emerging as a possible substitute for antibiotics. This study evaluated the therapeutic potency of a novel bacteriophage cocktail against MRSA mastitis. Two new bacteriophages (vB_SauR_SW21 and vB_SauR_SW25) with potent lytic activity against MRSA were isolated and characterized. The one-step growth curve displayed a rapid latent period (20–35 min) and substantial burst size (418 and 316 PFU/ cell). *In silico* analyses have confirmed the absence of antimicrobial resistance or virulence factor-encoding genes within their genomes. According to the results, combining these phages augmented their host range and virulence. The phage cocktail significantly reduced bacterial burden in a BALB/c mastitis model, demonstrating efficacy comparable to antibiotic treatment. Moreover, its administration led to decreased concentrations of IL-1β and TNF-α compared to the negative control group. The bacteriophage cocktail (SW21-SW25) exhibits a promising profile for therapeutic applications and may represent a novel substitute to antibiotics for managing MRSA bovine mastitis.

## Introduction

Methicillin-resistant *Staphylococcus aureus* (MRSA) is a principal pathogen in veterinary settings and public health [1]. It is the primary reason for healthcare-associated (HA-MRSA) and community-associated (CA-MRSA) infections, which can range from superficial to invasive infections [1, 2]. The World Health Organization (WHO) categorized this bacterium as a high-priority pathogen. These pathogens are notoriously hard to treat, cause substantial morbidity and mortality, develop resistance to most antibiotics, are challenging to prevent, spread rapidly, and have limited available treatment options [3]. Livestock-associated MRSA (LA-MRSA), affecting various domestic animals [1], poses a substantial global risk to both human and animal health [4]. Identified in the early 1970s, LA-MRSA serves as a substantial reservoir of MRSA outside hospital settings [1, 5].

Bovine mastitis is an inflammatory condition impacting the udder or mammary glands in cows [6] and has a high global prevalence. This infection causes a decline in the quality and abundance of the milk. Estimates suggest a 15% decline in milk yield per cow due to mastitis [4], with persistent effects preventing cows from reaching peak production throughout the rest of the lactation period [6]. Furthermore, mastitis leads to the slaughtering of chronically infected dairy cattle and increases costs for extra labor, medication, and veterinary services [7]. This widespread problem in the dairy industry [7] translates to substantial economic losses [8]. Moreover, milk from mastitis-infected cows poses a potential health risk due to harmful pathogens, heat-stable enterotoxins [9], antibiotic-resistance genes, and antibiotic residues [10]. Therefore, effective control and restorative strategies for bovine mastitis are crucial.

A recent meta-analysis reveals a concerning global prevalence of 4.3% (95% CI: 3.24–5.50) for bovine mastitis caused by MRSA. This infection poses a probable risk to veterinarians and farm workers, contributing to severe intra-herd infections [11]. Notably, MRSA strains exhibit not only *mecA*-mediated β-lactam resistance but also resistance to a broader spectrum of antibiotics. The emergence of multidrug-resistant (MDR) MRSA strains is remarkably alarming, as it hinders effective therapy and control. These MDR-MRSA strains often exhibit resistance to first-line antibiotics, rendering current treatment options limited [1]. This highlights the critical need to explore and develop alternative therapeutic strategies for effectively combating MRSA infections.

Bacteriophage therapy, employing viruses to cure bacterial infections, emerges as a promising antibiotic substitute [12]. Compared to conventional antibiotics, bacteriophages offer several advantages, including high host specificity [12, 13], rapid isolation of new phages, the potential for combining various bacteriophages (phage cocktails) for enhanced efficacy, and co-administration with antimicrobials to reduce antibiotic resistance [12]. While research has explored bacteriophages for bovine mastitis, reported phages have exhibited limitations in the host range. Therefore, research is ongoing to find more suitable bacteriophages for treating bovine mastitis [13]. This investigation aimed to evaluate the therapeutic potential of bacteriophages against MRSA-induced mastitis in a mouse model. Two MRSA bacteriophages were isolated from environmental sources and comprehensively characterized. The obtained biological data served as a foundation for subsequent *in vivo* experiments. Direct comparisons between phage and a conventional antibiotic were performed to estimate the clinical feasibility of phage therapy as a potential complementary or substitute treatment for bovine mastitis.

## Materials and methods

### Bacterial strains

A collection of 242 *S*. *aureus* isolates from milk samples of dairy cows afflicted with mastitis was considered for further examination. The isolates originated from the microbial collections of the veterinary faculties of Tehran University, Ferdowsi University of Mashhad, and Hamadan University. The collection period was from 2018 to 2022. Isolates were cultured on BHI agar (Merck, Germany), then incubated at 37°C for 24 hours. Afterward, the identity of the isolates was confirmed using Gram staining and conventional biochemical tests (catalase, coagulase, DNase, and mannitol fermentation) [14]. Verified *S*. *aureus* isolates were stored at -70°C as glycerol stocks.

### Detection of methicillin resistance by phenotypic method

Methicillin resistance was determined via the Kirby-Bauer disc agar diffusion test using a 30 μg cefoxitin disc based on Clinical and Laboratory Standard Institute (CLSI) guidelines [15]. *S*. *aureus* ATCC 43300 (MRSA) and *S*. *aureus* ATCC 25923 (MSSA) served as quality controls.

### Detection of methicillin resistance by molecular method

Confirmation of methicillin resistance in phenotypically resistant isolates was achieved using a *mecA* gene-specific PCR assay. Genomic DNA was extracted from isolates using a boiling method. Each 15 μl reaction mixture contained 7.5 μl of Taq 2× master mix (Ampliqon, Denmark), 0.5 μl of each forward and reverse primer (10 pmol/μl) (forward primer: F-TGGCCAATTCCACATTGTTTCG and the reverse primer: R-TCCAGGAATGCAGAAAGACCA), 2 μl of template DNA, and 4.5 μl of sterile distilled water. The following conditions were used to perform the amplification: initial denaturation at 95°C for 5 minutes, 30 cycles of denaturation at 95°C for 40 seconds, annealing at 60°C for 30 seconds, and extension at 72°C for 30 seconds, followed by a final extension at 72°C for 5 minutes. Finally, the PCR products were evaluated under UV light after running at 120 V for 45 minutes on a 1% agarose gel.

### Antibiotic susceptibility testing

The sensitivity of MRSA isolates to doxycycline (30 μg), clindamycin (2 μg), linezolid (30 μg), and trimethoprim-sulfamethoxazole (25 μg) was assessed using the Kirby-Bauer disc diffusion method, according to the CLSI criteria [15].

### Sample collection and processing for bacteriophage isolation

Thirty-eight samples were collected from various sources, which could potentially harbor MRSA-specific bacteriophages. These included dairy wastewater (3 samples), dry manure (1 sample), fresh cow feces (9 samples), hospital wastewater (5 samples from hospitals in Tehran—Imam Khomeini Hospital, Children’s Medical Center Hospital, and Yas Hospital), and municipal wastewater (20 samples from different regions of Tehran). Municipal wastewater samples were obtained with the approval of the Tehran Provincial Water and Sewerage Organization. Hospital wastewater samples were collected with the consent of the hospital director and in collaboration with the hospital’s Environmental Health Department. Arrangements were made with the farm manager to facilitate the collection of samples from the cattle farm. All samples were collected aseptically and transported under cold chain conditions to the laboratory.

For solid samples (e.g., manure, feces), 0.5 g was mixed with 4.5 ml of 1x LB broth (1:10 ratio). The mixture was thoroughly combined by inverting the falcon tube. The sample was then incubated for 2 hours in a shaking incubator at 37°C and 150 rpm to facilitate phage release. Next, the contents were filtered through filter paper, and the filtrate was collected for further analysis.

The sample (wastewater or processed solid sample suspensions) underwent centrifugation at 8,000 rpm for 15 minutes at 20°C to remove debris and impurities. The resulting supernatant was then passed through a 0.22 μm pore-size sterile filter to eliminate bacterial contamination and achieve sample sterilization. The final sterile filtrate was stored at 4°C [16].

### Bacteriophage enrichment

*Staphylococcus aureus* ATCC 43300, a standard MRSA strain, was the host bacterium for isolating MRSA-targeting phages. A pure bacterial culture was obtained by streaking the strain on BHI agar and incubating it overnight. Subsequently, one colony from a fresh bacterial culture was inoculated in 1x LB broth and incubated overnight at 37°C with shaking (150 rpm). In the following step, 0.1 ml of the overnight bacterial culture (adjusted to OD_600_ = 0.5) along with 10 ml of 2x LB broth (containing CaCl_2_ at a final concentration of 2 mM) and 10 ml of a filtered wastewater sample were mixed, and then incubated in a shaking incubator at 37°C and 150 rpm for 18 to 20 hours. After the specified time, the suspension was centrifuged at 8,000 rpm and 4°C for 15 minutes, and then the supernatant was filtered using a 0.22 μm pore-size filter to eliminate any remaining bacterial cells and sterilize the solution. The lysate (filtrate containing enriched phages) was then stored at 4°C [16].

### Screening of the lysates for lytic bacteriophages

The existence of lytic bacteriophages in the lysate was assessed by the spot assay. Briefly, 0.1 ml of an overnight culture of *S*. *aureus* ATCC 43300 (OD_600_ = 0.05) was mixed with molten top agar (LB broth with 0.7% (w/v) agar and 2 mM CaCl_2_) and overlaid onto solidified bottom agar (LB broth with 1.5% (w/v) agar). Following the top agar solidification, 10 μl of lysate was spotted on the center of the overlaid lawn. After incubation at 37°C for 24 hours, the plate was examined for clearing zones (plaques) indicative of lytic phage activity against MRSA [16].

### Bacteriophage isolation and purification

Isolation of individual bacteriophages was achieved using the double-layer agar (DLA) assay. Briefly, a ten-fold serial dilution (10^−1^ to 10^−9^) was prepared from the lysate-containing bacteriophages. Subsequently, 0.1 ml aliquots of each dilution and 0.1 ml of overnight culture of *S*. *aureus* ATCC 43300 (OD_600_ = 0.5) were mixed with molten top agar and overlaid onto solidified bottom agar plates. After incubation at 37°C for 18–24 hours, distinct plaques were observed. Then, a streak culture method was used to purify phage plaques. A single phage plaque was selected, gently picked with a sterile pipette tip, and transferred to a new plate for streaking out phages. Then, 0.1 ml of overnight culture of *S*. *aureus* ATCC 43300 (OD_600_ = 0.5) was added to 4 ml of top agar. After mixing, the mixture was gently spread on the bottom agar plate. This streaking and overlay process was repeated up to three times to ensure the isolation of pure phage clones [16].

### Bacteriophage titration

The phage titer was determined utilizing the DLA method, as described above. Following the incubation, plaque counts were recorded for each dilution plate. The plaque-forming unit (PFU) per ml (PFU/ml) was calculated by multiplying the plaque count by 10 and then dividing by the dilution factor. The titer of bacteriophages in the dilution where plaque counts ranged from 30 to 300 was reported as the titer of bacteriophages present in the stock [16].

### Host range determination

The host range of the isolated bacteriophages was determined using a spot assay. Thirty-four MRSA and 26 MSSA isolates recovered from milk samples of cows with mastitis were evaluated. The presence of plaques after incubation indicated bacterial sensitivity to the bacteriophages, while their absence signified resistance [16]. Phages that met the criteria of high titer production (> 10^8^ PFU/ml) and an appropriate host range were included in the study, and their properties were characterized.

### Assessment of bacteriophage virulence via efficiency of plating

The efficiency of plating (EOP) of the bacteriophages was determined using phage-sensitive clinical isolates identified by the spot test. The phage titer (PFU/ml) was measured for each sensitive isolate and the host strain (*S*. *aureus* ATCC 43300) by the DLA method. EOP was calculated by dividing the phage titer in the clinical isolate by the phage titer in the host strain [17]. Based on the EOP values, the bacteriophage was classified into the following groups: highly virulent (0.1 < EOP < 1.00), moderately virulent (0.001 < EOP < 0.099), avirulent but active (EOP < 0.001), and avirulent (no plaques detected) [18].

### Determination of optimal MOI for bacteriophage-host interactions

The optimal MOI of the bacteriophages was determined by infecting *S*. *aureus* ATCC 43300 with serially diluted phage preparations. Briefly, a 10-fold serial dilution of bacteriophage was prepared in 1x LB broth containing 2 mM CaCl_2_. Each dilution was combined with an equivalent volume of host strain culture (10^6^ CFU/ml). After co-incubation at 37°C with shaking for 15 minutes, the suspension was centrifuged for 10 minutes at 5,000 ×g to separate free phages from phage-bacteria complexes. The supernatant was discarded, and the pellet was resuspended in 1 ml of 1x LB broth with 2 mM CaCl_2_ and incubated for 4 hours at 37°C with shaking at 150 rpm. The bacteriophage titer in each sample was then determined using the DLA method. Finally, the bacteriophage titer was divided by the number of host bacteria (10^6^ CFU/ml), and the ratio of bacteriophage to the host cell, or bacteriophage MOI, was determined. The optimal MOI was identified as the phage-to-bacteria ratio that resulted in the highest phage titer [19]. The experiment was repeated twice.

### Transmission electron microscopy (TEM)

A high-titer bacteriophage suspension (10^10^ PFU/ml) was centrifuged at 25,000 ×g for 60 minutes to pellet the phage particles. The supernatant was discarded, and the pellet was resuspended in 1 ml of ammonium acetate buffer (0.1 M, pH 7.2). This centrifugation and resuspension step was repeated twice for further purification. Following the final wash, 950 μl of the supernatant was removed, leaving approximately 50 μl containing the concentrated phage pellet. For negative staining, 10 μl of the concentrated phage suspension was applied to a carbon-coated copper grid. After incubation at room temperature for 5 minutes, a drop of 2% uranyl acetate stain was added. The grid was incubated with the stain for one minute, followed by removing the excess with filter paper. The grid was then rinsed with distilled water and allowed to dry. The prepared TEM sample was examined using a Philips EM208S transmission electron microscope (Netherlands) at an accelerating 100 kV [16].

### Investigating bacteriophage stability

The stability of the bacteriophages was evaluated under different temperature and pH conditions.

Temperature stability: Bacteriophage suspension (10^9^ PFU/ml) was incubated at 37°C, 50°C, 60°C, and 70°C. Samples were collected at 5, 15, 30, and 60 minutes and after 24 hours of incubation. The phage titer was determined using the DLA method. Furthermore, phage stability at 4°C, 25°C, and -20°C was assessed at 1 and 24-hour time points.

pH stability: Bacteriophage suspension (10^9^ PFU/ml) in 1x LB broth with varying pH (3 to 11, adjusted with NaOH or HCl) was incubated at 37°C for one hour. The phage titer was then determined using the DLA technique. All experiments were conducted in duplicate [19].

### Longevity test

A 2 ml aliquot of bacteriophage lysate was maintained at 4°C for one year. The phage concentration was measured every six months using the DLA method.

### Adsorption assay

The phage adsorption rate was determined following the method of Kropinski et al. [20]. In summary, the host bacteria were cultured until they reached the logarithmic growth phase, then diluted to reach OD_600_ = 0.1–0.2. The bacterial concentration (CFU/ml) was calculated using the colony count method. Subsequently, 9 ml of bacterial suspension was mixed with 1 ml of phage suspension (10^7^ PFU/ml) to achieve an MOI of 0.01. The mixture was incubated at 37°C with shaking (60 rpm). Aliquots (50 μl) were withdrawn every minute, diluted with 1x LB broth (950 μl), vortexed for 10 seconds, then kept on ice until titration. Unbound phages in the supernatant were quantified after centrifugation (16,000 ×g for 10 minutes) using the DLA method. Additionally, the phage adsorption rate constant (k, ml/min) was calculated using the formula provided, where B is the initial bacterial density (CFU/ml) and t is the time taken for the phage titer to drop from P0 (initial phage concentration) to P (final phage concentration). In this study, 1x LB broth was used as a negative control.


$$k=\frac{2.3}{\text{B}t}\log\frac{\text{P}0}{\text{P}}$$

### One-step growth curve

We employed a one-step growth curve assay, with slight modifications from the method described by Wang et al. [21]. Briefly, 900 μl of an overnight host culture supplemented with CaCl_2_ (2 mM) was mixed with 100 μl of bacteriophage suspension (10^6^ PFU/ml). This mixture was incubated for 10 minutes at 37°C to facilitate phage adsorption. Following centrifugation (7,500 ×g for 5 minutes) to remove unbound bacteriophages, the pellet was resuspended in 1 ml of fresh 1x LB broth with CaCl_2_ (2 mM). The suspension was then incubated at 37°C with shaking (150 rpm). Aliquots (100 μl) were collected every 5 minutes for 90 minutes, and their phage titers were determined using the DLA assay.

### Unveiling the phage genome: Extraction, sequencing, and annotation

To decipher the genetic makeup of the isolated bacteriophages, their genomic DNA was extracted using the Phage DNA extraction kit (Norgen Biotek, Canada), following the manufacturer’s instructions. Whole genome sequencing was performed using Illumina technology (Novaseq PE150 platform) by Novogene (South Korea). The sequencing quality was assessed using the FastQC tool (Galaxy Version 0.74+galaxy0) [22] on the Galaxy server (https://usegalaxy.eu). Subsequently, *de novo* assembly was conducted using the Shovil (Spades) tool (Galaxy Version 1.1.0+galaxy2) [23]. The RAST server was employed to predict structural and functional annotation of the assembled phage genome [24], and the results were confirmed using BLASTP [25]. The Proksee server (https://proksee.ca/) was utilized for genome mapping and GC content analysis [26]. Potential tRNA genes were predicted using tRNAscan-SE (Galaxy Version 2.0.5) [27]. Phage life cycle determination and genome analysis were conducted using the Phage AI tool (https://www.phage.ai/) [28]. Antibiotic resistance genes, virulence factors, and toxins in phage genomes were detected using comprehensive databases, including CARD [29], ResFinder [30], and VFDB [31]. The isolated bacteriophage was named following the methodology proposed by Adriaenssens and Brister [32]. Easyfig software was utilized to conduct a comparative analysis of gene arrangement among the phages [33]. Genome-to-genome similarity and average nucleotide identity (ANI) were determined using the VIRIDIC tool. A similarity threshold exceeding 95% was established for species delineation, while a threshold of over 70% was applied for genus classification [34]. To understand the evolutionary relationships of the isolated bacteriophages with other known phages, a phylogenetic analysis was conducted using VICTOR (Virus Classification and Tree Building Online Resource) [35].

### *In vivo* phage therapy (mouse model)

#### Ethics declarations

All animal experiments were performed according to the ethical guidelines set by the Iranian Ministry of Health and Medical Education [36] and were approved by the Animal Welfare and Research Ethics Committee at Tehran University of Medical Sciences (approval number: IR.TUMS.SPH.REC.1400.274). Strict adherence to humane treatment guidelines was observed throughout the study, with every effort made to diminish animal suffering. Animals were closely monitored by laboratory staff twice daily throughout the experimental period. To minimize animal discomfort, we ensured continuous access to food, water, and soft bedding.

#### Preparation of bacterial suspension

A clinical MRSA isolate (RS35, isolated from bovine mastitis), susceptible to both bacteriophages, was used to induce mastitis in mice. An overnight culture of RS35 was prepared in 1x LB broth and subsequently centrifuged (15 minutes at 4,000 ×g) to obtain a bacterial pellet. The pellet was rinsed thrice with sterile PBS and resuspended in PBS to achieve a 0.5 McFarland standard turbidity (OD_600_ = 0.08–0.13) [37].

#### Preparation of Phage cocktail

The phage suspensions were subjected to sequential filtration (Amicon 100 kDa), centrifugation (5,000 ×g for 15 minutes), and sterilization (0.22 μm filter). Phage titers were determined using the DLA method. To create the phage cocktail, equal volumes of each phage suspension (standardized by titer) were combined in a sterile container and thoroughly mixed.

#### Bacteriophage therapy in mouse

The *in vivo* phage therapy was performed according to the method described by Geng et al. with some modifications [37]. Briefly, 36 female BALB/c mice, aged and weight-matched, were procured from the animal center of the Pasteur Institute of Iran. All mice had a recent parturition history and were lactating (10–14 days postpartum). After a 24-hour acclimation period, the mice were randomly classified into six groups, each consisting of six animals. Mice in group 1 were healthy controls (blank control) that received no injections. The mice in groups 2 to 5 were injected with MRSA strain RS35 (1.5 × 10^8^ CFU/ml) to induce mastitis, and four hours after intramammary infection induction were treated with sterile PBS (negative control), ceftiofur sodium (5 mg/kg) (positive control), phage cocktail-1 (1 × 10^10^ PFU/ml) (phage therapy 1), and phage cocktail-2 (1 × 10^9^ PFU/ml) (phage therapy 2). The phage concentrations selected for the study were based on the optimum MOI determined for each phage. Mice in group 6 were the phage control group that received an injection of the phage cocktail (1 × 10^10^ PFU/ml) without prior MRSA infection.

For the mastitis induction, mice were anesthetized with 100 μl of a ketamine/xylazine mixture (9:1) via intraperitoneal injection (27-gauge insulin syringe). Subsequently, a small incision was made near the tip of each teat, and a 25 μl inoculum of MRSA (1.5 × 10^8^ CFU/ml) was carefully injected into the teat canals of the L4 and R4 of the abdominal mammary glands (31-gauge insulin syringe). Four hours after mastitis induction, mice received a 25 μl injection of PBS, ceftiofur sodium, and phage cocktail (two concentrations), depending on their assigned groups.

#### Analyses of blood samples

Three mice per group were anesthetized with an intraperitoneal injection of ketamine/xylazine and euthanized by cervical dislocation at 24 and 48 hours post-treatment. Blood samples (1 ml) were obtained via cardiac puncture from two of the three euthanized mice in each group and sent to the laboratory for routine blood testing. Complete blood counts (CBCs) were performed on these samples, measuring white blood cells (WBC, 1000/μl), red blood cells (RBC, ml/μl), neutrophilic granulocytes (NE, 1000/μl), hemoglobin (HGB, g/dl), hematocrit (HCT, %), platelet count (1000/μl), and other relevant parameters [37].

#### Bacterial and phage counting

A portion of mammary glands (100 mg) were homogenized in 500 μl sterile PBS using a homogenizer. The homogenate was centrifuged at 15,000 rpm for 10 minutes at 4°C, and the supernatant was collected and transferred to a new microtube. To quantify bacterial colony count (CFU/g of mammary gland) and phage titer (PFU/g of mammary gland), two mammary gland homogenates were used in each group at both time points. Serial dilutions (10^−1^ to 10^−9^) of each sample were prepared in 1x LB broth. For bacterial colony counts, 10 μl aliquots from dilutions 10^−1^ to 10^−4^ were plated on BHI agar and incubated at 37°C for 24 hours. Colony-forming units were then enumerated. In groups treated with the phage cocktail (groups 4, 5, and 6), plaque-forming units (PFU) were also determined from the mammary gland extracts using the DLA technique [37].

#### Measurement of cytokines

Cytokine concentrations (IL-1β and TNF-α) were quantified in mammary gland homogenates from two mice per group at each time point using Enzyme-linked immunosorbent assay (ELISA) [37]. Assays were performed according to the manufacturer’s protocols (Mabtech, Sweden).

### Statistical analysis

Statistical analysis was performed using GraphPad Prism version 8.4.3. The one-way and two-way ANOVA tests were used to compare group means, followed by Tukey’s test for multiple comparisons. Furthermore, a t-test was used for specific comparisons, such as phage titers under different temperature and pH conditions. Error bars in the graphs represent the standard error of the mean (± SEM). A P-value ≤ 0.05 was considered statistically significant.

## Results

### Identification and susceptibility testing of MRSA isolates

We examined the frequency of MRSA in milk samples of cows with mastitis and tested their resistance to different antibiotics. Among the 242 isolates collected, 147 were verified as *S*. *aureus*, all originating from subclinical mastitis. A concerning proportion (36/147, 24.5%) of these *S*. *aureus* isolates were identified as MRSA, among which 94.44% (N = 34) harbored the *mecA* gene. Table 1 shows the antibiotic susceptibility patterns of MRSA isolates to four different antibiotics. Clindamycin displayed the highest resistance rate (79%), followed by moderate resistance to trimethoprim-sulfamethoxazole (35%). Conversely, low resistance rates were observed for linezolid (0%) and doxycycline (3%).

**Table 1:** The antibiotic susceptibility pattern of MRSA isolates.

Antibiotics	Isolates, N (%)		
	Susceptible	Intermediate	Resistant
Doxycycline	26 (76.5)	7 (20.5)	1 (3)
Trimethoprim-sulfamethoxazole	22 (65)	0	12 (35)
Clindamycin	6 (18)	1 (3)	27 (79)
Linezolid	34 (100)	0	0

### Isolation of bacteriophages

**Fig 1:**
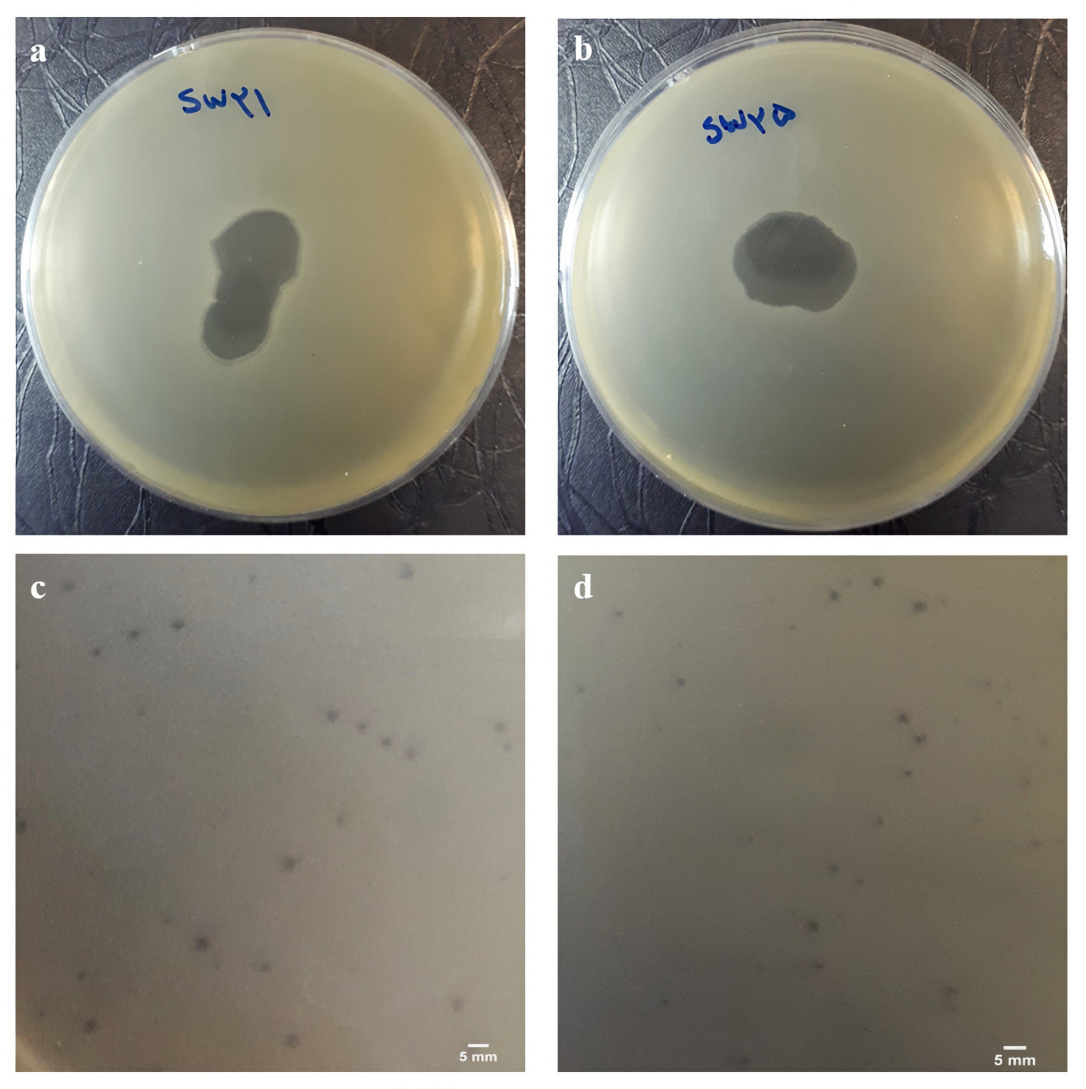
**Positive spot tests**, representing the presence of MRSA lytic bacteriophages in the lysates (a, b). **Plaque morphology of bacteriophages**. Staphylococcus phage vB_SauR_SW21 (c) and Staphylococcus phage vB_SauR_SW25 (d). Scale bar, 5 mm.

We isolated two bacteriophages with specific lytic activity against MRSA from municipal and hospital wastewaters (Fig 1A and 1B). The phages were reproducible and exhibited a high titer (> 10^8^ PFU/ml) in the DLA test. They formed small, apparent, round plaques (1–2 mm diameter) (Fig 1C and 1d). We designated the phages as Staphylococcus phage vB_SauR_SW21 and Staphylococcus phage vB_SauR_SW25, respectively.

### Characteristics of bacteriophages

#### Morphology in electron microscopy

**Fig 2:**
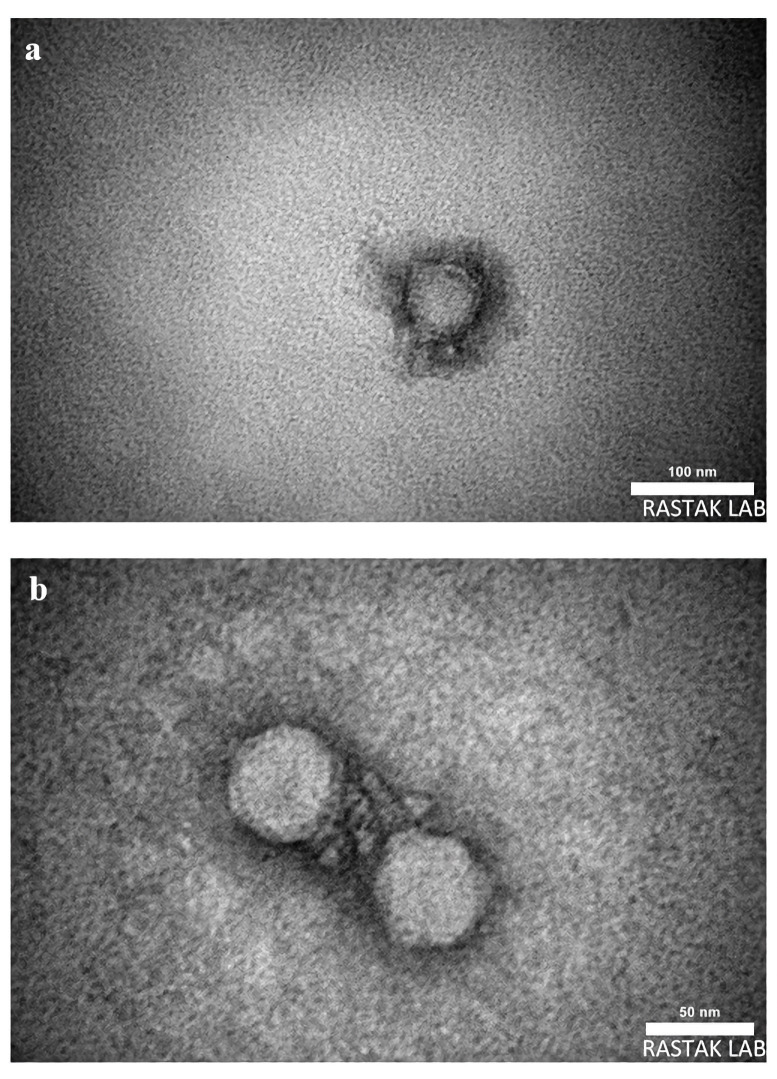
Electron micrographs of bacteriophages. (a) Staphylococcus phage vB_SauR_SW21 and (b) Staphylococcus phage vB_SauR_SW25.

TEM images demonstrated that phages vB_SauR_SW21 and vB_SauR_SW25 possessed icosahedral capsids measuring 51.2 ± 2 nm and 53 ± 2 nm, respectively. Both phages had short, non-contractile tails of 31.9 ± 2 nm and 32.3 ± 2 nm length (Fig 2). These morphological characteristics are consistent with phages belonging to the family *Podoviridae*.

#### Host range

The host ranges of bacteriophages were established by spot assay on 34 *mecA*-positive MRSA and 26 MSSA bovine mastitis isolates. Phage vB_SauR_SW21 lysed 29.4% (10/34) of MRSA isolates, while vB_SauR_SW25 lysed 65% (22/34). A phage cocktail (an equimolar mixture of two bacteriophages) demonstrated enhanced lysis, affecting 68% (23/34) of MRSA isolates. Against MSSA, vB_SauR_SW21 achieved a lysis rate of 73% (19/26), compared to 61.5% (16/26) for vB_SauR_SW25. The phage cocktail exhibited the highest efficacy, lysing 92% (24/26) of MSSA isolates.

#### Efficiency of plating (EOP)

EOP for phage vB_SauR_SW21 ranged from 0 to 0.75, while those for vB_SauR_SW25 varied from 0.0001 to 4. The phage cocktail showed a broader EOP range of 0.001 to 10. Phage vB_SauR_SW21 demonstrated high virulence against 70% (7/10) of isolates, whereas vB_SauR_SW25 was highly virulent against 27.2% (6/22) of isolates. The phage cocktail significantly enhanced virulence, with high efficacy against 56.5% (13/23) of isolates. The EOP findings are detailed in Table 2.

**Table 2:** EOP of phage vB_SauR_SW21, phage vB_SauR_SW25, and phage cocktail (SW21-SW25).

		vB_SauR_SW21		vB_SauR_SW25		Phage cocktail	
No.	Strain name	EOP	Classification	EOP	Classification	EOP	Classification
1	RS8	-	-	0.5	Highly virulent	5	Highly virulent
2	RS10	0.001	Moderately virulent	0.1	Highly virulent	1	Highly virulent
3	RS13	0.35	Highly virulent	0.0001	avirulent but active	5	Highly virulent
4	RS14	0.1	Highly virulent	0.005	Moderately virulent	2.6	Highly virulent
5	RS19	0.5	Highly virulent	0.1	Highly virulent	3	Highly virulent
6	RS20	-	-	0.4	Highly virulent	0.5	Highly virulent
7	RS26	0.75	Highly virulent	0.006	Moderately virulent	5	Highly virulent
8	RS28	0.4	Highly virulent	0.022	Moderately virulent	10	Highly virulent
9	RS29	-	-	0.3	Highly virulent	2	Highly virulent
10	RS30	-	-	0.05	Moderately virulent	0.5	Highly virulent
11	RS33	0.75	Highly virulent	0.0005	avirulent but active	4	Highly virulent
12	RS34	-	-	0.01	Moderately virulent	0.1	Highly virulent
13	RS35	0.75	Highly virulent	0.6	Highly virulent	0.6	Highly virulent
14	RS6	0	avirulent	0.01	Moderately virulent	0.001	Moderately virulent
15	RS17	-	-	0.005	Moderately virulent	0.001	Moderately virulent
16	RS18	0	avirulent	-	-	0.001	Moderately virulent
17	RS22	-	-	0.0001	avirulent but active	0.001	Moderately virulent
18	RS23	-	-	0.0001	avirulent but active	0.001	Moderately virulent
19	RS27	-	-	0.0001	avirulent but active	0.001	Moderately virulent
20	RS31	-	-	0.0001	avirulent but active	0.001	Moderately virulent
21	RS32	-	-	0.0001	avirulent but active	0.001	Moderately virulent
22	RS36	-	-	0.0001	avirulent but active	0.001	Moderately virulent
23	RS3	-	-	0.0001	avirulent but active	0.001	Moderately virulent

#### Optimal MOI

We investigated the optimum multiplicity of infection (MOI) for both phages. The analysis demonstrated that bacteriophage titers for vB_SauR_SW21 and vB_SauR_SW25 peaked at an MOI of 10, as shown in S1 Fig. These findings were statistically significant compared to other MOI values (*P* ≤ 0.0001).

#### Thermal stability

**Fig 3:**
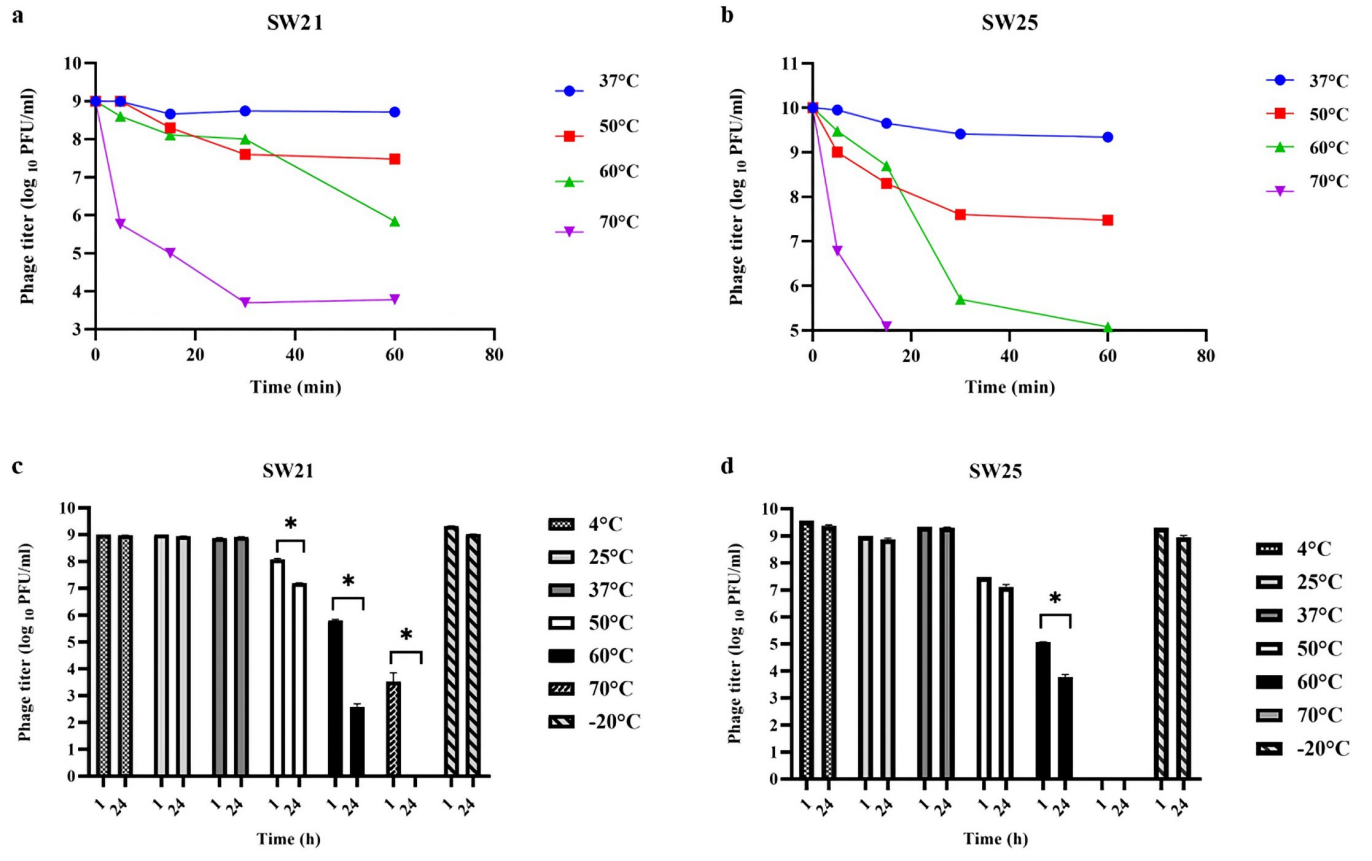
Stability of Staphylococcus phage vB_SauR_SW21 and Staphylococcus phage vB_SauR_SW25 at various temperatures. (a, b) bacteriophages stability at different temperatures during 60 minutes of incubation (at 5-, 15-, 30-, and 60-minute intervals). (c, d) bacteriophages stability at different temperatures after 1 and 24 hours of incubation. The results are presented as means ± standard errors (SEM) based on two separate experiments. The asterisk (*) at the top of the columns signifies a statistically significant discrepancy (*P* ≤ 0.05) among the groups’ values.

The thermal stability of phages vB_SauR_SW21 and vB_SauR_SW25 was evaluated at various temperatures. Phage vB_SauR_SW21 demonstrated stability at 4°C, 25°C, 37°C, and -20°C for both 1 and 24 hours, with no significant changes in phage titer (*P* > 0.05). However, the phage exhibited heat sensitivity, with a marked decrease in titer at 50°C and 60°C (*P* ≤ 0.05) and complete inactivation at 70°C (Fig 3A and 3C). In contrast, phage vB_SauR_SW25 showed temperature-dependent stability. While maintaining stability at 4°C, 25°C, and -20°C, it experienced an initial decline followed by stabilization at 37°C. At 50°C, a gradual decline in titer was noticed, and complete inactivation occurred at 70°C (Fig 3B and 3D). These results indicate differential thermal stability profiles for the two phages.

#### pH stability

**Fig 4:**
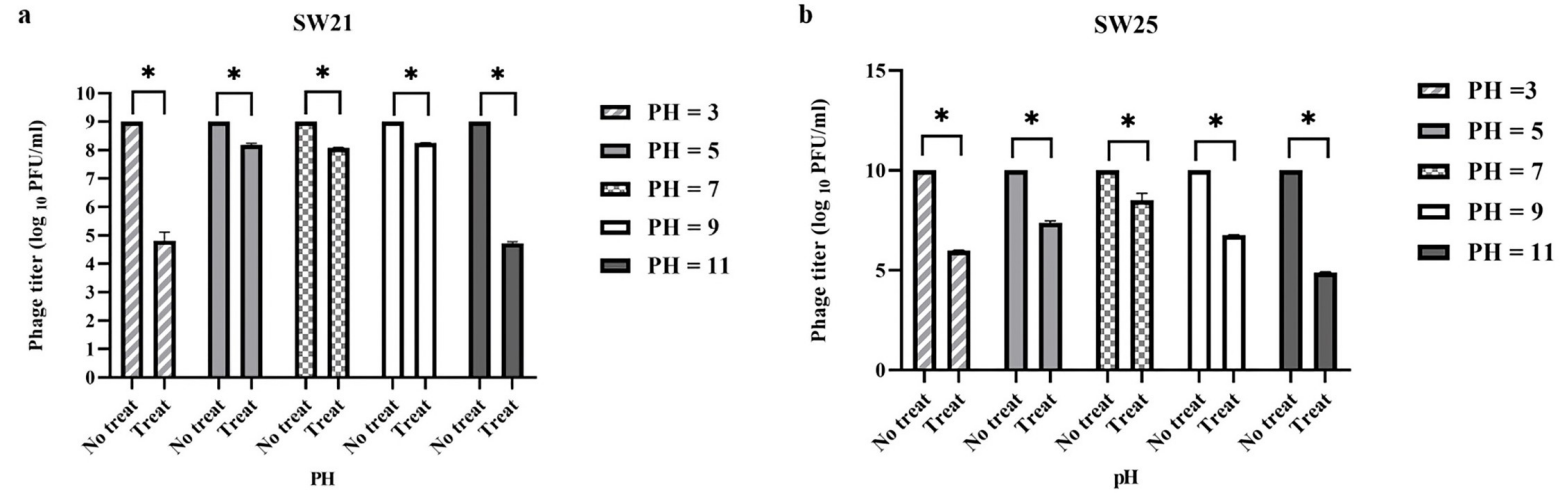
Stability of Staphylococcus phage vB_SauR_SW21 (a) and Staphylococcus phage vB_SauR_SW25 (b) at various pHs. The results are presented as means ± standard errors (SEM) based on two separate experiments. The asterisk (*) at the top of the columns signifies a statistically significant discrepancy (*P* ≤ 0.05) among the groups’ values.

Phage vB_SauR_SW21 demonstrated moderate pH stability, with consistent, yet statistically significant, titer reductions of 0.7–0.9 logs at physiological pH levels (5, 7, and 9, *P* ≤ 0.05). However, the phage exhibited significantly higher sensitivity (*P* ≤ 0.0001) to extreme pH conditions (3 and 11), experiencing a substantial 4-log titer decrease (Fig 4A). In contrast, phage vB_SauR_SW25 displayed limited pH stability overall. While a statistically significant titer reduction of log 2.3 was observed at pH 7 (*P* = 0.0181), more pronounced decreases of 4 and 6 logs occurred at pH 3 and 11, respectively (*P* ≤ 0.05) (Fig 4B).

#### Longevity test

To evaluate the stability of the isolated bacteriophages during storage, we monitored their titers at 4°C for a year, measuring every six months (S2 Fig). Phage vB_SauR_SW21 experienced a 0.4 log titer reduction over this period (from 2.2 × 10^9^ PFU/ml to 8.8 × 10^8^ PFU/ml); however, this decrease was not statistically meaningful (*P* = 0.0588). While this indicates some titer reduction, it’s still within an acceptable range for many practical applications. Conversely, phage vB_SauR_SW25 showed remarkable stability, maintaining its initial titer of 1 × 10^9^ PFU/ml for six months and only experiencing a slight reduction to 8.6 × 10^8^ PFU/ml after a year (*P* = 0.1914). Based on these findings, both phages demonstrate reasonable stability at 4°C, making this temperature suitable for long-term storage.

#### Adsorption rate

The adsorption kinetics of phages vB_SauR_SW21 and vB_SauR_SW25 were assessed. The results revealed rapid phage adsorption to host cells, with over 95% of phages attaching within 4 and 6 minutes, respectively (S3 Fig). The adsorption rate constant was calculated to be 2.12 × 10^−8^ ml/min and 4.32 × 10^−8^ ml/min for vB_SauR_SW21 and vB_SauR_SW25, respectively, signifying a high affinity between the phages and their target host.

#### One-step growth curve

**Fig 5:**
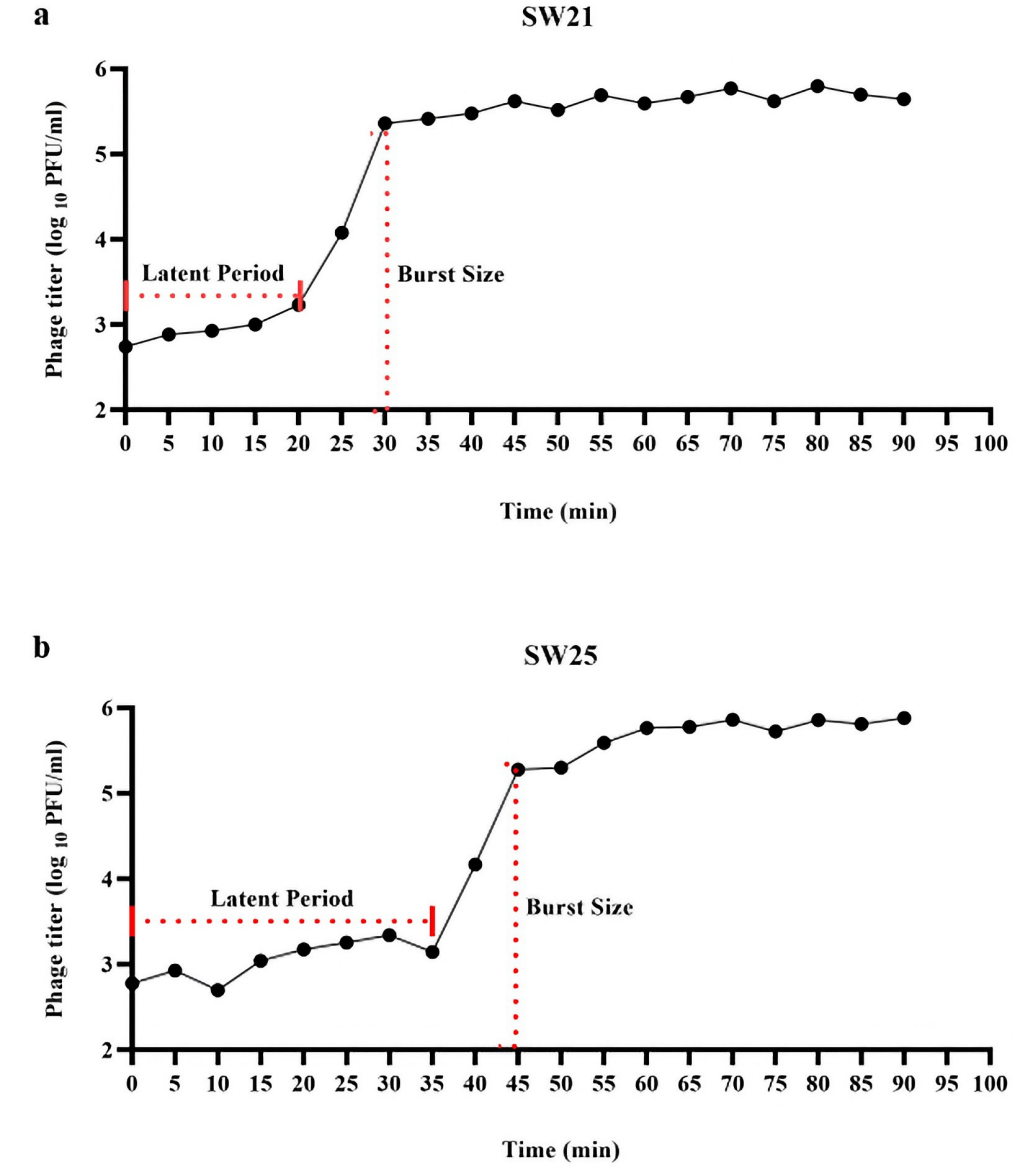
The one-step growth curve for Staphylococcus phage vB_SauR_SW21 (a) and Staphylococcus phage vB_SauR_SW25 (b). The x-axis displays the incubation period for the phages in conjunction with the host *S*. *aureus* ATCC 43300. The y-axis indicates the log_10_ value of plaque-forming units of phage per 1 ml (PFU/ml).

The one-step growth curve (Fig 5) illustrates the phages’ replication cycle. For phages vB_SauR_SW21 and vB_SauR_SW25, the latent period, the duration between bacteriophage adsorption and the release of new viruses, were 20 and 35 minutes, respectively. Furthermore, the burst size, defined as the average number of new virion phages made per infected cell, was calculated to be 418 and 316 PFU/cell, respectively.

#### Genomic features of bacteriophages

**Fig 6:**
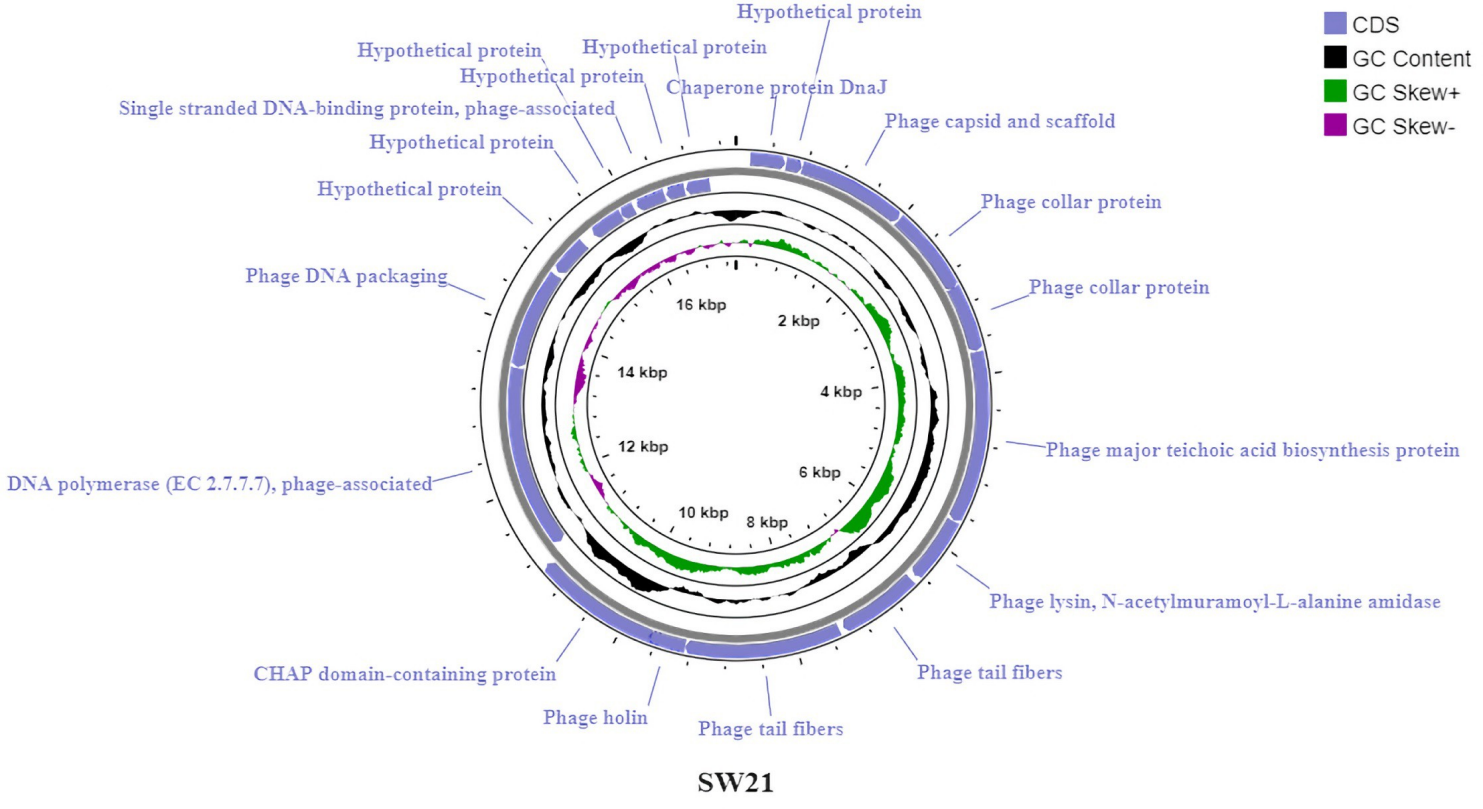
Genetic maps of Staphylococcus phage vB_SauR_SW21 plotted by Proksee server.

The genome of Staphylococcus phage vB_SauR_SW21 was sequenced and assembled into a single, complete sequence of 17,369 bp using the Spades software. The assembled genome was a linear double-stranded DNA (dsDNA) molecule with a relatively low G+C content of 29.5%. The genome comprised 19 coding sequences (CDS), with 58% of the genes oriented in the positive (sense) direction. The ratio of coding regions to the total genome was 93.79%. Interestingly, only one CDS (5.3%) utilized the TTG start codon, while the remaining 94.7% initiated translation with the more common ATG codon. Two types of stop codons were detected in the CDSs, including TAA (78.95%) and TAG (21.05%). The CDSs on the genome were categorized into various functional groups, including those implicated in the synthesis of the phage’s structural components (capsid, collar, and tail proteins), packaging of the bacteriophage genome, phage DNA synthesis (DNA polymerase enzyme and single-stranded DNA-binding protein), membrane lysis (holin) and host cell wall degradation (lysin), and a set of genes with unknown functions labeled as hypothetical proteins (Fig 6). More details regarding the CDSs of this phage are outlined in the S1 Table.

**Fig 7:**
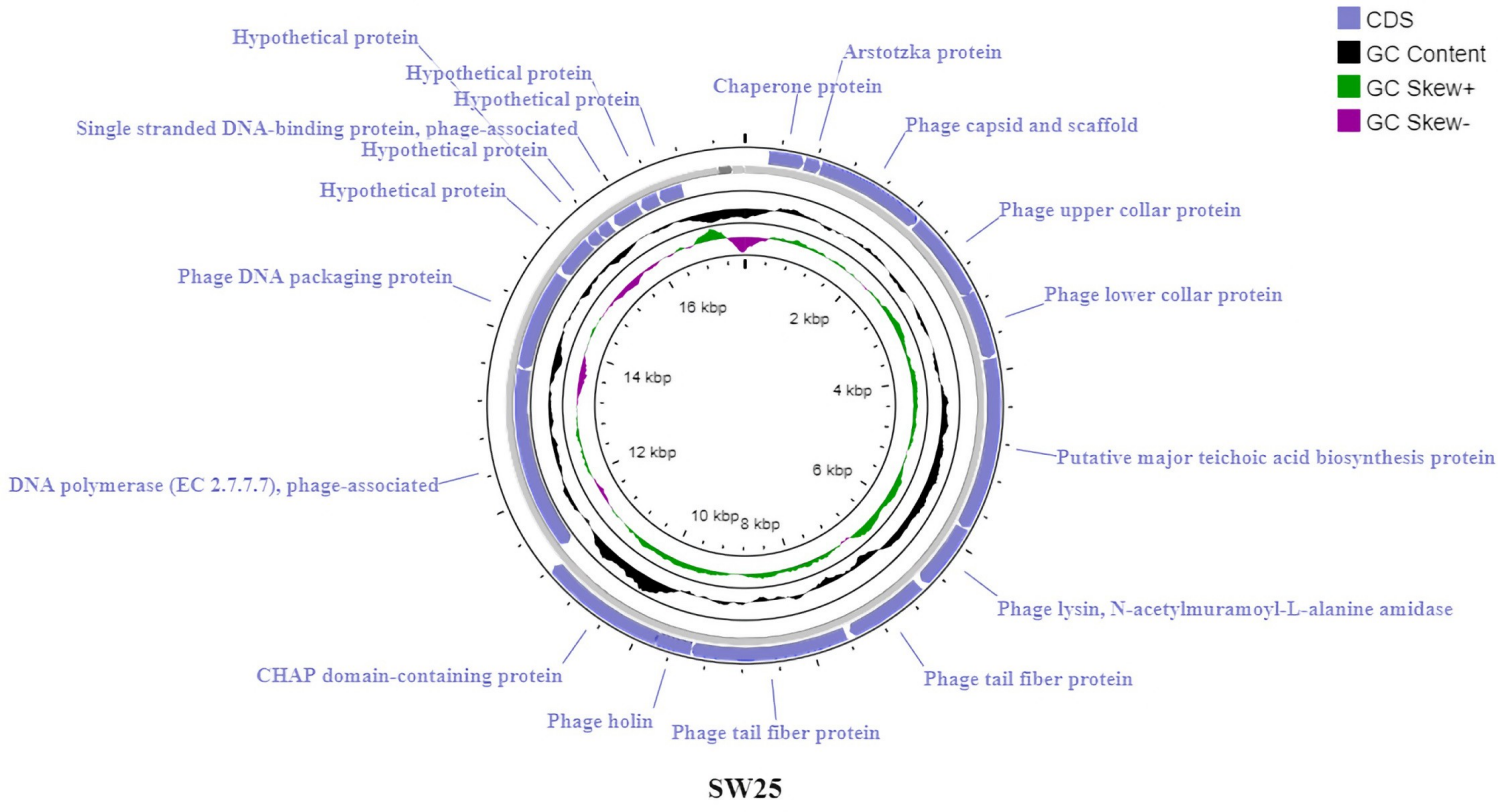
Genetic maps of Staphylococcus phage vB_SauR_SW25 plotted by Proksee server.

Bioinformatics analysis of phage vB_SauR_SW25 revealed a linear dsDNA genome (17,223 bp) with a G+C content of 29.24%. The genome encoded 19 proteins, with 58% of genes (11/19) located on the positive strand, similar to phage vB_SauR_SW21. Coding regions comprised 93.16% of the genome, initiating exclusively with ATG start codons and terminating with TAA (78.95%) or TAG (21.05%) stop codons. Predicted protein functions include structural components, genome packaging, DNA replication, host cell lysis, and release. A subset of proteins remained uncharacterized and were classified as hypothetical (Fig 7). Detailed gene annotations are provided in the S2 Table.

The tRNA scan SE software did not detect tRNA-encoding genes. The phage genomes underwent scrutiny in multiple databases such as CARD, ResFinder, and VFDB to check for antibiotic resistance genes, virulence determinants, and plasmids, with no genes associated with these aspects were identified.

Comparative genomic analysis using Easyfig revealed a substantial resemblance in gene content and arrangement between phages vB_SauR_SW21 and vB_SauR_SW25 (S4 Fig). While overall genomic architecture is conserved, notable differences are concentrated in the terminal regions. Average nucleotide identity (ANI) analysis using VIRDIC software revealed a 92.1% similarity between phages vB_SauR_SW21 and vB_SauR_SW25 (S5 Fig). Based on the ANI threshold of 70% for genus-level classification and 95% for species-level delineation, these phages are considered members of the same genus (*Rosenblumvirus*). Analysis using the Phage AI server revealed the lytic nature of both phages. Supporting this lytic character, no genes associated with lysogeny (e.g., integrase, excisionase) were identified in their genomes. Moreover, the predicted taxonomy for the phages was the family *Podoviridae* and the genus *Rosenblumvirus*. In a significant taxonomic revision implemented by the Bacterial and Archaeal Viruses Subcommittee (BAVS) in August 2022, the order *Caudovirales* was abolished along with its constituent families: *Siphoviridae*, *Myoviridae*, and *Podoviridae*. The replaced classification now employs the Caudoviricetes class, which encompasses all bacterial and archaeal phages characterized by icosahedral capsid symmetry, dsDNA genomes, and the presence of tails [38]. Leveraging the NCBI Taxonomy database, the comprehensive lineage classification of both bacteriophages is established as follows: Viruses, Duplodnaviria, Heunggongvirae, Uroviricota, Caudoviricetes, *Rountreeviridae*, *Rakietenvirinae*, *Rosenblumvirus*.

The genus *Rosenblumvirus* encompasses 46 characterized bacteriophages specific to *S*. *aureus*. However, only 22 of these phages have been classified into 16 distinct species, while the remaining 24, including our phages vB_SauR_SW21 and vB_SauR_SW25, remain unclassified [39].

According to the ViralZone resource [40], phages within the genus *Rosenblumvirus* are generally non-enveloped viruses and exhibit a head-tail morphology. The capsid has an approximate diameter of 55 nm and likely displays T = 4 icosahedral symmetry. The non-contractile tail is approximately 27 nm long and features a collar adorned with 12 appendages at the neck region, which are crucial for recognizing and entering host cells. Furthermore, Rosenblumviruses harbor linear dsDNA genomes ranging from 16 to 20 kb in size, encoding approximately 20 to 30 genes [41]. These features are consistent with the properties of the phages identified in this experiment.

#### Phylogenetic analysis of bacteriophages

To elucidate the evolutionary relationship between these phages, VICTOR software was employed to conduct a phylogenetic analysis incorporating publicly available phage genomes from GenBank (S6 Fig). The maximum likelihood phylogenetic tree revealed that phage vB_SauR_SW21 shared the highest genetic relatedness with phage 351Saur083PP, which is a specific *S*. *aureus* phage belonging to the genus *Rosenblumvirus* isolated in Poland [42]. Furthermore, phage vB_SauR_SW21 displayed significant genetic similarity to phage GRCS (NCBI Reference Sequence: NC_023550.1) [43], a reference phage in the genus *Rosenblumvirus*, and phages Simurgh and Huma originating from Iran [44]. Phage vB_SauR_SW25 also exhibits genetic relatedness to the phages mentioned above, albeit to a lesser extent than vB_SauR_SW21. Additional details regarding the genomic characteristics of these phages can be found in the S3 Table. These phages exhibit a significant similarity regarding their genome size, number of CDS, GC content, and protein functions.

### *In vivo* efficacy of bacteriophage cocktail

#### CFU and PFU assessments

**Fig 8:**
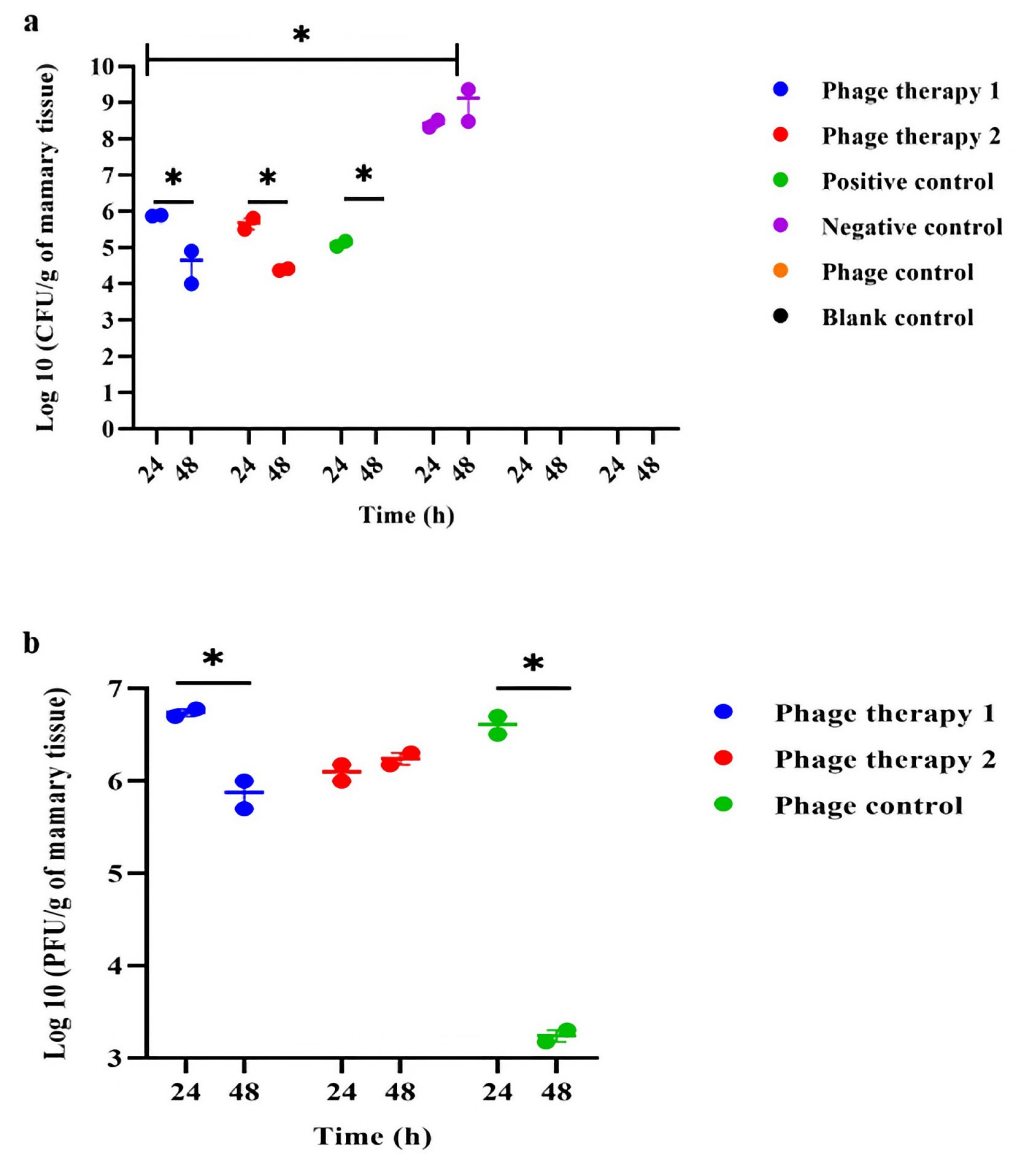
Comparison of bacterial titers (a) and phage titers (b) in different groups. Phage therapy 1: mastitis + phage cocktail-1 (1 × 10^10^ PFU/ml, MOI = 100), phage therapy 2: mastitis + phage cocktail-2 (1 × 10^9^ PFU/ml, MOI = 10), positive control: mastitis + ceftiofur sodium, negative control: mastitis + PBS, phage control: healthy mice + phage cocktail (1 × 10^10^ PFU/ml), blank control: healthy mice without treatment. The bar represents means ± SEM from duplicate experiments. Statistically significant discrepancies (*P* ≤ 0.05) between group means are signified by an asterisk (*).

To evaluate the efficacy of our bacteriophage cocktail (SW21-SW25) in treating MRSA-induced mastitis, bacterial burden (CFU/g of mammary tissue) and phage titer (PFU/g of mammary tissue) were determined for each experimental group. As shown in Fig 8A, the positive control group demonstrated a significant reduction in bacterial load after 48 hours of ceftiofur sodium treatment (*P* = 0.0229), indicating effective antibiotic therapy. Both phage therapy groups (1 and 2) exhibited significant decreases in bacterial titer between 24 and 48 hours (*P* ≤ 0.05). Compared to the initial inoculum (1 × 10^8^ CFU/ml), phage cocktail-1 (MOI = 100) achieved a 4.3 log reduction at 48 hours. Similarly, phage cocktail-2 (MOI = 10) resulted in 4.6 log reductions after 48 hours of treatment. While both phage concentrations effectively reduced bacterial burden, no significant discrepancy was observed between them (*P* > 0.05). Although ceftiofur sodium induced a more pronounced bacterial reduction than either phage cocktail (*P* ≤ 0.05), the overall outcomes reinforce the prospect of the phage cocktail as a fortunate control and therapeutic strategy for MRSA-induced mastitis.

Phage titer analysis (Fig 8B) revealed a significant reduction in phage numbers within the phage control group at the 48-hour time point (*P* = 0.045). A similar, though less pronounced, decrease was observed in phage therapy group 1 (*P* = 0.013), whereas phage titers in group 2 remained relatively constant (*P* > 0.05).

#### Blood test

**Fig 9:**
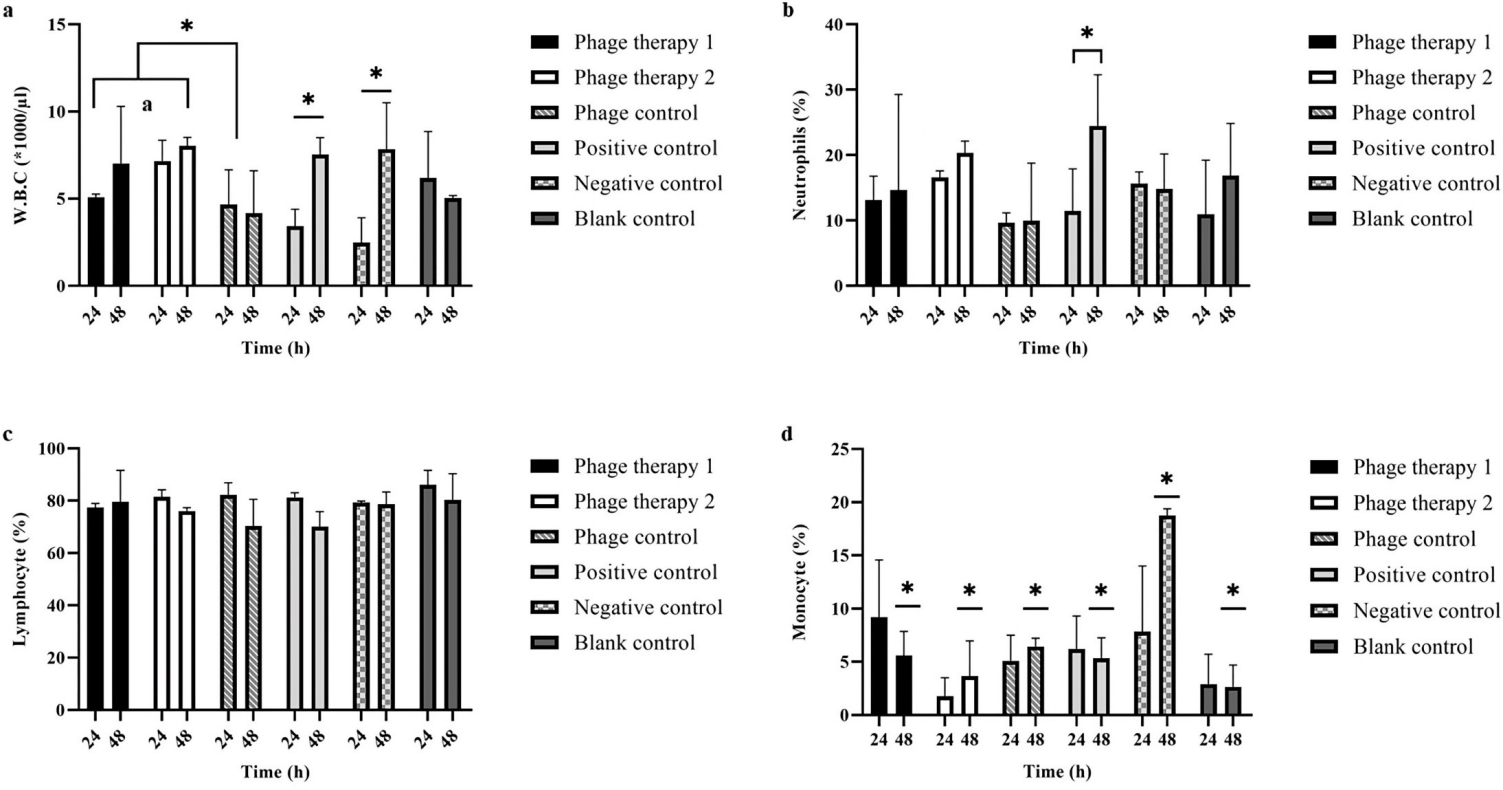
Comparison of the particular blood cell parameters across the various murine treatment groups. Phage therapy 1: mastitis + phage cocktail-1 (1 × 10^10^ PFU/ml, MOI = 100), phage therapy 2: mastitis + phage cocktail-2 (1 × 10^9^ PFU/ml, MOI = 10), phage control: healthy mice + phage cocktail (1 × 10^10^ PFU/ml), positive control: mastitis + ceftiofur sodium, negative control: mastitis + PBS, blank control: healthy mice without treatment. The bar represents means ± SEM from duplicate experiments. Statistically significant discrepancies (*P* ≤ 0.05) between group means are signified by an asterisk (*).

The analysis of blood cell counts demonstrated that mice administered with phage cocktail-2 exhibited increased mean white blood cell (WBC) levels compared to the other experimental groups (Fig 9). While the phage control group exhibited stable WBC levels at 24 and 48 hours, these counts were substantially less than those in phage therapy groups (*P* ≤ 0.05). The differentiation of WBC subtypes showed that neutrophil counts remarkably increased in the positive control group at the 48-hour mark (24.5%). Lymphocytes constituted the predominant WBC subtype across all experimental groups (mean 70–85%), with the highest proportion observed in the blank control at 24 hours (86.05%). Notably, monocyte levels were significantly elevated in the negative control group at 24 hours (18.75%) compared to other groups. Comprehensive analysis of additional blood parameters (S4 Table) revealed no significant alterations in red blood cell count (RBC), hemoglobin, hematocrit, or other related indices (MCV, MCH, MCHC, RDW-CV, RDW-SD) across all groups (*P* > 0.05). Similarly, platelet counts and associated parameters (PDW, MPV, P-LCR, PCT) remained unaffected. A comparison of these results to established normal ranges for BALB/c mice indicated that all samples fell within expected reference values.

#### Changes of TNF-α and IL-1β concentrations

**Fig 10:**
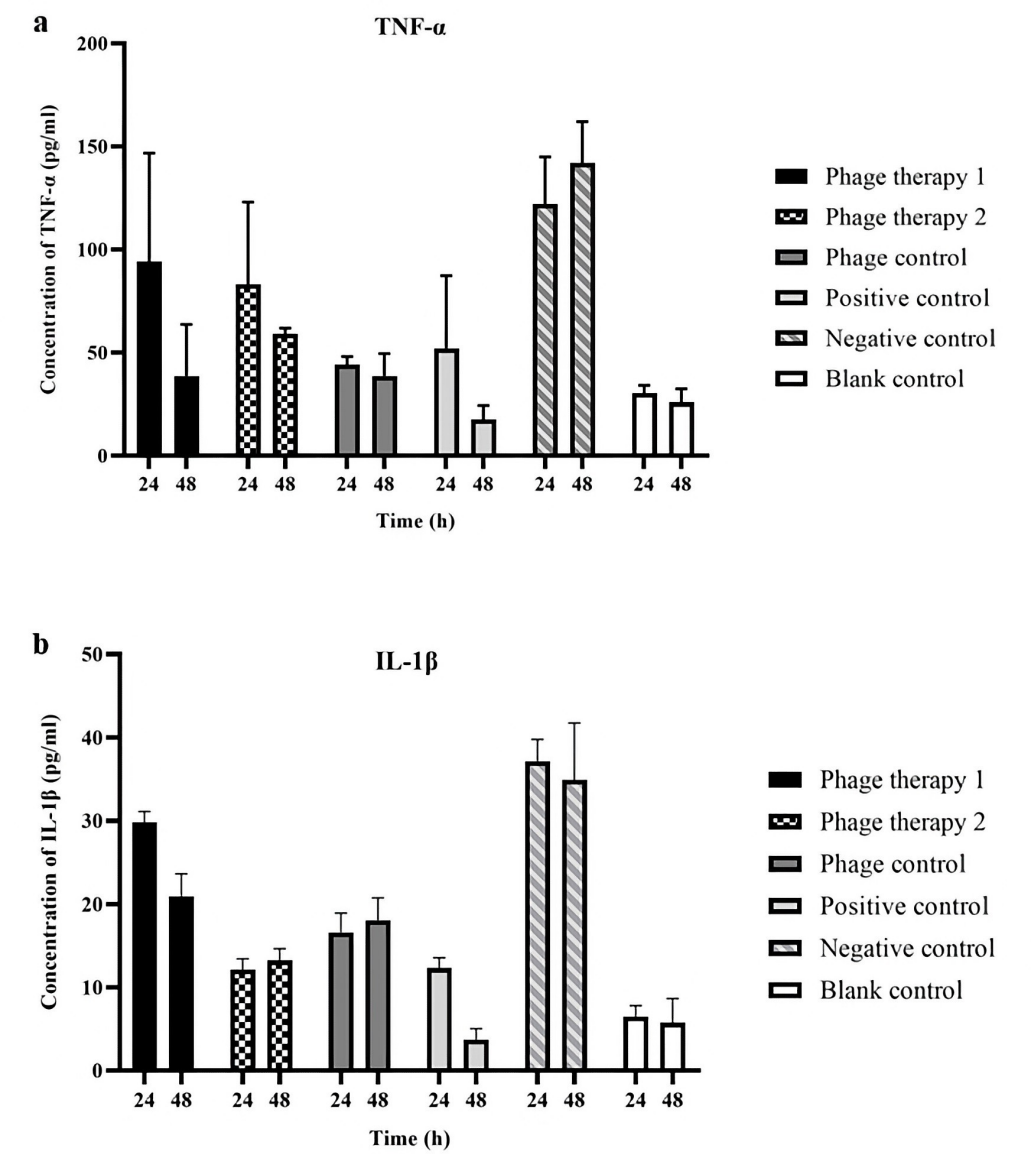
The concentration of TNF-α (a) and IL-1 β (b) following phage treatment in a murine mastitis model. Phage therapy 1: mastitis + phage cocktail-1 (1 × 10^10^ PFU/ml, MOI = 100), phage therapy 2: mastitis + phage cocktail-2 (1 × 10^9^ PFU/ml, MOI = 10), phage control: healthy mice + phage cocktail (1 × 10^10^ PFU/ml), positive control: mastitis + ceftiofur sodium, negative control: mastitis + PBS, blank control: healthy mice without treatment. The bar represents means ± SEM from duplicate experiments.

Pro-inflammatory cytokine concentrations were evaluated in mammary gland tissues using ELISA. As shown in Fig 10, relative to the negative control group, all treatment groups (positive control and phage therapy groups 1 and 2) displayed significant reductions in IL-1β and TNF-α concentrations, with the positive control group (ceftiofur sodium-treated) exhibiting the most pronounced decrease (*P* ≤ 0.05). Among the phage-treated groups, phage cocktail-2 significantly reduced IL-1β levels, while cocktail-1 achieved a more evident reduction of TNF-α. Unlike the healthy control, the phage control group (healthy mice receiving a phage cocktail) showed elevated levels of pro-inflammatory cytokines, indicating a mild inflammatory response to the phages.

## Discussion

Bovine mastitis, a multifaceted inflammatory disease of the mammary glands, arises from a complex interplay of environmental, host-related, and pathogenic factors. Researchers have extensively linked *S*. *aureus*, a ubiquitous opportunistic pathogen, to clinical and subclinical mastitis in dairy cattle. This bacterium significantly impacts economic viability by causing reductions in milk yield and quality. Furthermore, bovine *S*. *aureus* isolates are recognized as principal contributors to foodborne diseases [45], potentially contaminating the human food chain through consuming infected milk. Accordingly, consuming unpasteurized dairy products in specific regions presents a considerable public health concern [46]. Currently, treating MRSA bovine mastitis is increasingly complicated due to the high antibiotic resistance of the bacterium and the necessity for prolonged antibiotic therapy to achieve a successful bacteriological cure. Furthermore, the growing prevalence of MDR strains within this pathogen population further diminishes the efficacy of standard antibiotic regimens [45]. Consequently, developing novel therapeutic strategies to complement or replace antibiotics for MRSA bovine mastitis has become a top research priority globally.

MRSA strains resist all β-lactam antibiotics due to altered penicillin-binding proteins (PBPs), known as PBP2a or PBP2′. These modified PBPs function differently from normal PBPs and have a reduced affinity for β-lactams. The genes encoding these proteins, *mecA* and *mecC* (the latter sharing 69% homology with *mecA*), are located on mobile genetic elements (MGEs) referred to as staphylococcal cassette chromosome *mec* (SCC*mec*) [4]. In this investigation, *S*. *aureus* isolates were collected from bovine mastitis, and their methicillin resistance was evaluated. The findings revealed that 36 out of 147 isolates (24.5%) were MRSA, with 94.44% (34 isolates) harboring the *mecA* gene. Notably, the results obtained from phenotypic methods (cefoxitin sensitivity testing) and genotypic methods (detecting the *mecA* gene by PCR) did not align in identifying methicillin-resistant strains. This discrepancy was also reported in other studies [46, 47] and may be due to mutations or truncations in the *mecA* gene, leading to reduced gene expression. Additionally, errors in phenotypic testing, such as improper culture conditions or operator mistakes, could contribute to false results. Moreover, homologous genes like *mecC* might not be detected by *mecA*-specific primers, resulting in a negative molecular test despite a methicillin-resistant phenotype [46].

Analysis of the antibiotic susceptibility patterns of the 34 MRSA strains revealed linezolid (0%) and doxycycline (3%) as the most effective antibiotics, exhibiting the lowest resistance rates. These findings regarding clindamycin and trimethoprim-sulfamethoxazole resistance diverge from those reported by Khazaie et al. [46]. In their study, MRSA isolates demonstrated minimal resistance to clindamycin (0%) and a lower prevalence of trimethoprim-sulfamethoxazole resistance (18.18%). Similar to our findings, a study by Egyptian researchers documented complete susceptibility (0% resistance) of bovine mastitis-associated MRSA isolates to linezolid. However, this investigation observed no resistance to trimethoprim-sulfamethoxazole (0%) among the isolates, contrasting with our observed moderate resistance rate of 35% [48]. The dairy industry heavily relies on antibiotics to treat and prevent mastitis. However, when implemented excessively or without adherence to proper protocols, antibiotic therapy can contribute to the rise of antimicrobial-resistant (AMR) bacterial pathogens. AMR has evolved into a worldwide public health concern due to the misuse of antibiotics, which has led to the emergence of highly resistant "superbug" bacteria unsusceptible to many, if not all, available antimicrobial agents. The potential for AMR bacteria to disseminate through the food chain poses a further complication in effectively managing infectious illnesses in humans and animals [49]. A critical strategy for mitigating the dissemination of AMR strains involves continuous evaluation of antibiotic resistance patterns within mastitis-causing isolates across diverse geographical areas. This ongoing surveillance would result in the development of evidence-based treatment strategies tailored to the specific resistance profiles observed in each region [50].

Our investigation resulted in isolating two novel Staphylococcus-specific phages, vB_SauR_SW21 and vB_SauR_SW25, exhibiting lytic activity against MRSA strains. Both phages are categorized as *Podovirus* due to their distinctive morphological characteristics and demonstrated broad host ranges, successfully lysing 10 and 22 MRSA isolates, respectively, and 19 and 16 MSSA isolates. Their ability to lyse MRSA and MSSA strains suggests a potential advantage for *S*. *aureus* infection treatment. This finding further implies the utilization of a common receptor by the phage for bacterial attachment and invasion. A confluence of factors affects the spectrum of bacterial hosts that a bacteriophage can infect, referred to as its host range. These factors encompass host-related characteristics (active and passive defense mechanisms), environmental conditions (temperature and pH), and features encoded by the phage (receptor-binding proteins (RBPs)) [51]. Despite these limitations, phages possess counter-defense mechanisms, overcoming host resistance and broadening their host range [52]. One strategy to exploit this adaptability and enhance the effectiveness of phage therapy involves using phage cocktails, which combine multiple phages with different host ranges [53]. In our study, combining two bacteriophages have markedly expanded the host spectrum and enhanced the virulence of the bacteriophages.

As bacteriophages are increasingly used in therapeutic applications, there is an escalating concern about the development of phage-resistant bacterial strains. Studies have shown that when a single phage is used repeatedly over an extended period, bacteria can evolve resistance to that particular phage [54]. Bacteria have various mechanisms to develop resistance against phage therapy, such as spontaneous mutations (blocking phage adsorption through the alteration or loss of bacterial receptors), superinfection resistance mechanisms (inhibition of phage DNA entry), restriction-modification (RM) and CRISPR/Cas systems (degradation of phage DNA), abortive viral replication systems (inhibition of phage replication, transcription, or translation), cyclic oligonucleotide-mediated phage defense mechanisms, and MGEs [55, 56]. Researchers believe that employing phage cocktails may be a beneficial approach to prevent the evolution of phage resistance in bacteria. By carefully selecting phages with diverse properties and using cocktails, the levels of pathogenic bacteria are significantly reduced, enabling the patient’s immune system or other antibacterial therapies to eradicate the infection [57].

Environmental factors like temperature and pH significantly influence bacteriophage interactions with their bacterial hosts. These factors encompass the incidence of bacteriophage infections, the longevity of stored phages (stability), and the success of phage infection (efficacy). Exposure to extreme fluctuations in temperature and pH can damage a phage’s structure or genetic material, potentially compromising its functionality [58]. While phages exhibit remarkable tolerance to a broad spectrum of environmental stressors, most phages targeting human pathogens prefer optimum temperature and pH conditions, mirroring their bacterial hosts [58]. Understanding these factors governing phage stability is crucial for optimizing phage therapy applications and maximizing their potential as antimicrobial agents. Our investigation into the stability of Staphylococcus phage vB_SauR_SW21 and Staphylococcus phage vB_SauR_SW25 revealed optimal viability at temperatures of 4°C, 25°C, 37°C, and -20°C. A substantial decline in titer was observed at temperatures of 50°C and 60°C, indicating that the bacteriophages’ viability is adversely affected by heat. Moreover, complete inactivation of the phages occurred at 70°C. The phages exhibited relative stability at pH 7 while demonstrating vulnerability at pH extremes 3 and 11. These findings indicate that the phages retain considerable viability under temperature and pH conditions resembling those of the human and animal body (37°C and pH 7.4).

The phages in this study exhibited good stability when stored at 4°C for 12 months. The ability of phage preparations to maintain their effectiveness over time is essential for both successful phage therapy and regulatory approval. A prospected phage for treatment should have enough longevity and preserve its activity without significant loss of potency during handling or storage. This feature is necessary because decreased potency can reduce the effectiveness of the treatment. Various methods have been developed to enhance phage stability, such as spray-drying, lyophilization, extrusion-drying, and emulsion techniques. However, the effectiveness of these methods varies depending on the specific phage type and the formulation used (e.g., liquid, gel, powder) [56].

The latent period and burst size are critical parameters for comprehending phage-bacteria interactions and selecting suitable phages for therapeutic applications [59]. A rapid exponential growth rate of phages is desirable for efficient host bacterial infection and necessitates a larger burst size, shorter generation time, and faster phage adsorption. However, burst size and generation time are intricately associated with the latent period. Prolonged latent periods are typically associated with bigger burst sizes, while shorter latent periods are linked to faster generation times. Optimizing the latent period of phages thus presents a challenge due to this trade-off between maximizing burst size and minimizing generation time [60]. Ideally, a phage for therapeutic purposes would possess both a quick latent period and a big burst size [61]. Our research demonstrated that phages vB_SauR_SW21 and vB_SauR_SW25 exhibit a favorable profile, with a latent period of 20 and 35 minutes and a substantial burst size of 418 and 316 PFU/cell, respectively. These characteristics suggest their lytic nature and potential suitability for bacteriophage therapy applications.

Whole genome sequencing is an indispensable tool for safeguarding the use of bacteriophages in therapeutics. It enables confirmation of the lack of deleterious genes within the bacteriophage genome, such as those encoding virulence factors or antimicrobial resistance determinants. Furthermore, this technique underpins the precise classification of novel phages and elucidates their evolutionary relationships with previously identified phages. According to bioinformatics analyses, phages vB_SauR_SW21 and vB_SauR_SW25, belonging to the genus *Rosenblumvirus*, had a similar G+C content and genomic organization to previously described phages of this genus [43, 44].

Bacteriophages are vital for horizontal gene transfer (HGT) among bacterial strains through transduction [62]. Their capacity to carry antibiotic resistance and virulence-encoding genes poses a potential concern for phage therapy. Recommended criteria for the isolation and characterization of suitable phages include the preference for non-temperate (lytic) phages, which exhibit a limited ability to engage in generalized transduction. Lytic phages are less prone to transduction since they typically degrade the host’s DNA to utilize its components for replication. This process significantly reduces the likelihood of generalized transduction by limiting the availability of host DNA during the packaging of viral DNA. To confirm that the selected phages are non-temperate, phenotypic (e.g., plaque morphology) and genotypic (e.g., absence of integrase genes) assessments should be performed. Additionally, phages should be evaluated for their capacity to transfer antibiotic-resistance genes or by identifying host DNA within their genomes [63]. Fortunately, the isolated bacteriophages in this study exhibited a strictly lytic life cycle devoid of genes associated with lysogeny, a favorable characteristic for therapeutic applications. Moreover, their genome analysis demonstrated that the phages did not contain any genes encoding for bacterial virulence factors or antimicrobial resistance determinants. These combined genomic features position these bacteriophages as encouraging candidates for the phage therapy of MRSA infections.

The efficacy of bacteriophage therapy hinges on the safety of the phage preparations, which poses significant challenges in manufacturing and formulation. To be widely used in medicine, phage production must be on large scales according to Good Manufacturing Practices (GMP) regulations. While phage production complies with the strict regulations typically applied to pharmaceuticals to guarantee high-quality standards, there are currently no dedicated policies for bacteriophage manufacturing. Researchers have established quality and safety standards for sustainable bacteriophage therapy products to address the lack of precise guidelines for phage manufacturing. One principal requirement is avoiding phages that carry genes for lysogeny, virulence, or antibiotic resistance. Additionally, phage preparations should be free from impurities like endotoxins or contain them below a certain level [56]. The presence of endotoxins in phage preparations can result in significant systemic side effects if not effectively eliminated, making this a critical consideration for intravenous administration [64]. Researchers have developed various purification methods to remove these harmful components from phage preparations, but none have achieved perfect results. Since bacteriophages are biological entities, developing reliable manufacturing processes that adhere to GMP regulations is necessary to prevent inconsistencies between phage preparations. Another key factor is quality control for phage stock solutions, which involves regular checks for longevity (shelf life), sterility, cytotoxicity, and pH levels. Recent advancements in bacteriophage production have reignited interest in phage therapy in Western nations. However, significant hurdles remain before phage therapy can achieve broad regulatory approval [56].

Phage therapy assessment using the mastitis mouse model demonstrated that both phage therapy groups (after 48 hours) significantly reduced bacterial titers by log 4.3 and 4.6, respectively (*P* ≤ 0.05). While the phage cocktail did not eradicate the MRSA load observed with ceftiofur sodium, it demonstrated significant efficacy in controlling the infection. Phages and bacteria engage in a dynamic predator-prey relationship. As such, complete eradication of the target bacterial population using phage therapy alone is often unattainable. However, phages can significantly reduce bacterial load, enabling the host’s immune system to effectively clear any remaining infection [37].

Our study observed increased WBC counts in phage therapy groups compared to the phage control, suggesting a potential immune response to MRSA infection or phage treatment. However, the lower WBC count in the phage control group indicates a minimal immune response to the phage cocktail alone. Conversely, Geng and colleagues noted that the mean WBC count in the negative control group was three times greater than that in the other treatment groups. While both phage and ceftiofur sodium treatment groups exhibited lower WBC counts than the negative control, their levels remained elevated relative to the healthy control group [37]. The immune system is essential in identifying and eliminating bacteriophages within animal and human bodies [56]. Bacteriophages have an immunogenic composition consisting of genetic material (DNA or RNA) and a protein coat that renders them recognizable to the immune system [55]. The reticuloendothelial system (RES), mainly found in the liver and spleen, is the principal mechanism responsible for the clearance of phages. This system comprises phagocytic cells, like monocytes and macrophages, which engulf and destroy these foreign invaders [65]. The presence of antibodies considerably hinders the effectiveness of bacteriophage therapy, as they can attach to and neutralize phages, thereby inhibiting their ability to infect bacterial cells [65, 66]. Moreover, the immune response mediated by T-cells is essential in combating viral infections. Antigen-presenting cells (APCs) can activate CD4+ T-helper cells upon phage uptake, resulting in the formation of memory cells and the production of specific neutralizing antibodies [65]. Consequently, individuals with pre-existing high levels of antibodies against phages may experience reduced efficacy of phage therapy due to accelerated clearance of the phages. This antibody production could arise from prior exposure to phages, whether through previous treatments or natural encounters. Additionally, the complement system further aids in eliminating phages from the body, highlighting the comprehensive role of the immune response [66]. Therefore, the phage-specific immune response is an influential factor in developing phage cocktails for therapeutic applications [67].

Acute mastitis is characterized by inflammation, marked by an influx of inflammatory cells and proteins. The pro-inflammatory cytokines IL-1β and TNF-α are crucial for immune responses and can also induce tissue damage when overproduced. Concentrations of IL-1β and TNF-α were measured employing ELISA to assess mammary gland inflammation. Our findings revealed significantly elevated IL-1β and TNF-α concentrations in the negative control group, indicative of a vigorous inflammatory response to MRSA infection. In contrast, the positive control group displayed a marked reduction in cytokine levels after 48 hours of ceftiofur sodium treatment, suggesting effective suppression of inflammation and bacterial infection. Additionally, phage therapy groups exhibited lower cytokine levels than the negative control, indicating a potential anti-inflammatory effect. However, cytokine levels were elevated in the phage control group relative to the healthy control, indicating that the phage components triggered an immune response, likely due to their antigenic properties. Reducing the phage concentration might lessen the inflammatory reaction. Future studies should explore a broader range of phage concentrations to determine the optimal balance between infection control and reduced inflammation.

A prominent obstacle in phage therapy for bovine mastitis is delivering phages to inflamed tissues, as they do not possess chemotactic movement to reach bacteria in distant locations, and the udder’s large and intricate structure complicates this process [68]. Similar to other studies [37, 69], we used intramammary injection to deliver phage cocktails. The research by Iwano et al. [68] included intravascular and intraperitoneal injections besides intramammary administration. Utilizing a mouse model of mastitis, they found that both intravenous and intraperitoneal delivery of phage ΦSA012 effectively reduced the multiplication of *S*. *aureus* in the mammary glands. However, they also observed that when phages were administered intravenously or intraperitoneally without accompanying bacteria, they were rapidly cleared from the bloodstream and mammary tissue within four hours due to elimination by macrophages [68]. Consequently, intramammary injection appears to be the most suitable method for administering phages in mastitis treatment, as it delivers the bacteriophage solution directly to the affected mammary gland. This approach facilitates high local concentrations of phages essential for targeting and eradicating the specific pathogenic bacteria involved in the infection.

Bovine mastitis can be exacerbated by biofilm formation. Pathogens responsible for this infection can congregate within extracellular polymeric substances (EPS), leading to biofilm development [70]. These biofilms resemble chronic biofilm infections seen in humans and different animals, often resulting in persistent and recurrent cases of mastitis [71]. Biofilm-embedded bacteria exhibit heightened resistance to immune system components [72] and are significantly less vulnerable to antimicrobial agents than their planktonic forms. This enhanced tolerance plays a crucial role in the chronic nature of the infection [73]. Moreover, biofilms serve as hotspots for HGT, promoting the spread of antibiotic-resistance genes [74]. The proximity of cells in the EPS matrix and the prolonged retention time enhance the efficiency of genetic exchange processes [74], including conjugation and transformation [72]. Consequently, the existence of biofilms in mammary glands diminishes the effectiveness of antibiotic therapy, making persistent infections problematic to eradicate. Several pathogens, including *S*. *aureus*, coagulase-negative staphylococci (CNS), *Escherichia coli*, *Enterococcus faecalis*, *Streptococcus uberis*, *S*. *dysgalactiae*, and *S*. *agalactiae*, employ biofilm formation as a critical virulence factor in bovine mastitis [73]. Phage therapy is a promising strategy to combat biofilms [75] and has primarily been studied in laboratory settings against single-species biofilms associated with mastitis. However, multispecies biofilms are more common in natural conditions [71]. To fully evaluate the efficacy of bacteriophage therapy for chronic mastitis, *in vivo* studies that assess the impact of phage cocktails (such as SW21-SW25 cocktail) on these complex biofilm communities within the mammary glands are necessary.

This study has some limitations. First, we did not delve into phage therapy’s long-term efficacy and safety. Future research will be essential to ascertain whether the therapeutic effects and inflammatory responses to phages remain consistent. Second, we did not explore the potential synergistic effects of phages and antibiotics. Researching combined phage and antibiotic treatments could provide practical insights into the benefits of using phages to combat antibiotic-resistant bacteria.

## Conclusion

Our study represents the first investigation of its type in Iran, addressing a gap in the literature. We successfully isolated two bacteriophages (Staphylococcus phage vB_SauR_SW21 and Staphylococcus phage vB_SauR_SW25) from the local environment, which exhibit a broad host spectrum against MRSA bovine strains prevalent in our country. This is critical, as phage effectiveness can be strain-specific, and locally sourced phages may offer enhanced therapeutic outcomes for treating endemic infections. Additionally, our comprehensive evaluation unveiled that the phage cocktail containing these phages was efficacious and harmless for treating mastitis caused by MRSA in mice. This suggests that our cocktail could be a practical alternative to antibiotics in managing MRSA bovine mastitis.

## Supporting information

S1 Fig The optimal MOI of Staphylococcus phage vB_SauR_SW21 (a) and Staphylococcus phage vB_SauR_SW25 (b). Results are displayed as means ± SEM from duplicate experiments.1: MOI = 1000, 2: MOI = 100, 3: MOI = 10, 4: MOI = 1, 5: MOI = 0.1, 6: MOI = 0.01, 7: MOI = 0.001, 8: MOI = 0.0001, 9: MOI = 0.00001.
(TIF)

S2 Fig Stability of Staphylococcus phage vB_SauR_SW21 and Staphylococcus phage vB_SauR_SW25 at 4°C for a year. Results are displayed as means ± SEM from duplicate experiments.
(TIF)

S3 Fig Adsorption rate of Staphylococcus phage vB_SauR_SW21 (a) and Staphylococcus phage vB_SauR_SW25 (b) to S. aureus ATCC 43300. The x-axis shows the incubation time (minutes) of phages with the host bacterium, while the y-axis displays the percentage of free (unadsorbed) phages.
(TIF)

S4 Fig Comparative genomic analysis of phages vB_SauR_SW21 and vB_SauR_SW25 using Easyfig. (TIF)

S5 Fig The ANI values of phages vB_SauR_SW21 and vB_SauR_SW25 calculated by VIRIDIC software. (DOCX)

S6 Fig Phylogenetic tree of Staphylococcus phage vB_SauR_SW21 and Staphylococcus phage vB_SauR_SW25 plotted by VICTOR software. (DOCX)

S1 Table Genome annotation of Staphylococcus phage vB_SauR_SW21. (DOCX)

S2 Table Genome annotation of Staphylococcus phage vB_SauR_SW25. (DOCX)

S3 Table Characteristics of some closely related Staphylococcus phages of the genus *Rosenblumvirus*. (DOCX)

S4 Table Blood parameters in different mice groups after 24 and 48 hours. (DOCX)

S1 Data Raw data of bacterial strains isolated from milk samples of bovine mastitis (36 MRSA and 26 MSSA). (XLSX)

S2 Data Raw data of experiments performed for characterization of Staphylococcus phage vB_SauR_SW21 (host range, EOP, optimal MOI, thermal stability, pH stability, longevity, adsorption rate, and one-step growth curve). (XLSX)

S3 Data Raw data of experiments performed for characterization of Staphylococcus phage vB_SauR_SW25 (host range, EOP, optimal MOI, thermal stability, pH stability, longevity, adsorption rate, and one-step growth curve). (XLSX)

S4 Data Raw data of experiments performed on phage cocktail (SW21-SW25) (host range and EOP). (XLSX)

S5 Data Raw data of experiments performed in animal study (CFU determination, PFU determination, specific blood cell parameters (white blood cell, lymphocytes, neutrophils, and monocyte), TNF-α and IL-1β concentrations). (XLSX)

## References

[1] Algammal AM, Hetta HF, Elkelish A, Alkhalifah DHH, Hozzein WN, Batiha GE-S, et al. Methicillin-Resistant Staphylococcus aureus (MRSA): one health perspective approach to the bacterium epidemiology, virulence factors, antibiotic-resistance, and zoonotic impact. Infect Drug Resist. 2020:3255–65. doi: 10.2147/IDR.S272733 33061472 PMC7519829

[2] Kourtis AP, Hatfield K, Baggs J, Mu Y, See I, Epson E, et al. Vital signs: epidemiology and recent trends in methicillin-resistant and in methicillin-susceptible Staphylococcus aureus bloodstream infections—United States. MMWR. 2019;68(9):214. doi: 10.15585/mmwr.mm6809e1 30845118 PMC6421967

[3] Jesudason T. WHO publishes updated list of bacterial priority pathogens. Lancet Microbe. 2024. doi: 10.1016/j.lanmic.2024.07.003 39079540

[4] Khairullah AR, Ramandinianto SC, Effendi MH. A review of livestock-associated methicillin-resistant Staphylococcus aureus (LA-MRSA) on bovine mastitis. Syst Rev Pharm. 2020;11(7):172–83.

[5] Crespo-Piazuelo D, Lawlor PG. Livestock-associated methicillin-resistant Staphylococcus aureus (LA-MRSA) prevalence in humans in close contact with animals and measures to reduce on-farm colonisation. Ir Vet J. 2021;74:1–12. 34362463 10.1186/s13620-021-00200-7 PMC8348836

[6] Sharun K, Dhama K, Tiwari R, Gugjoo MB, Iqbal Yatoo M, Patel SK, et al. Advances in therapeutic and managemental approaches of bovine mastitis: a comprehensive review. Vet Q. 2021;41(1):107–36. doi: 10.1080/01652176.2021.1882713 33509059 PMC7906113

[7] Titze I, Lehnherr T, Lehnherr H, Krömker V. Efficacy of bacteriophages against Staphylococcus aureus isolates from bovine mastitis. Pharmaceuticals. 2020;13(3):35. doi: 10.3390/ph13030035 32110980 PMC7151712

[8] Angelopoulou A, Warda AK, Hill C, Ross RP. Non-antibiotic microbial solutions for bovine mastitis–live biotherapeutics, bacteriophage, and phage lysins. Crit Rev Microbiol. 2019;45(5–6):564–80. doi: 10.1080/1040841X.2019.1648381 31403343

[9] Kaoud HA. Mini-Review: Alternative Therapies of Bovine Mastitis. EJAE. 2015;2(9):23–6.

[10] Ashraf A, Imran M. Causes, types, etiological agents, prevalence, diagnosis, treatment, prevention, effects on human health and future aspects of bovine mastitis. Anim Health Res Rev. 2020;21(1):36–49. doi: 10.1017/S1466252319000094 32051050

[11] Zaatout N, Hezil D. A meta‐analysis of the global prevalence of methicillin‐resistant Staphylococcus aureus (MRSA) isolated from clinical and subclinical bovine mastitis. J Appl Microbiol. 2022;132(1):140–54. doi: 10.1111/jam.15192 34171143

[12] Titze I, Krömker V. Antimicrobial activity of a phage mixture and a lactic acid bacterium against Staphylococcus aureus from bovine mastitis. Vet Sci. 2020;7(1):31. doi: 10.3390/vetsci7010031 32155751 PMC7157551

[13] El-Sayed A, Kamel M. Bovine mastitis prevention and control in the post-antibiotic era. Trop Anim Health Prod. 2021;53:1–16. doi: 10.1007/s11250-021-02680-9 33788033

[14] Mahon CR, Lehman DC. Textbook of diagnostic microbiology-e-book. 7th ed. St. Louis, Missouri: Elsevier Health Sciences; 2022.

[15] PA. W. CLSI Performance Standards for Antimicrobial Susceptibility Testing. CLSI Document, supplement M 100, 31st ed. USA: Clinical Laboratory Standards Institute (CLSI); 2021.

[16] Clokie M, Kropinski A. Bacteriophages: Methods and protocols, volume 1: Isolation, characterization, and interactions. Methods in molecular biology, USA: Humana press;2009. 69–81 p.

[17] Khan Mirzaei M, Nilsson AS. Isolation of phages for phage therapy: a comparison of spot tests and efficiency of plating analyses for determination of host range and efficacy. PLoS one. 2015;10(3):e0118557. doi: 10.1371/journal.pone.0118557 25761060 PMC4356574

[18] Gibson SB, Green SI, Liu CG, Salazar KC, Clark JR, Terwilliger AL, et al. Constructing and characterizing bacteriophage libraries for phage therapy of human infections. Front Microbiol. 2019;10:2537. doi: 10.3389/fmicb.2019.02537 31781060 PMC6861333

[19] Kwiatek M, Parasion S, Mizak L, Gryko R, Bartoszcze M, Kocik J. Characterization of a bacteriophage, isolated from a cow with mastitis, that is lytic against Staphylococcus aureus strains. Arch Virol. 2012;157:225–34. doi: 10.1007/s00705-011-1160-3 22045271

[20] Kropinski AM. Measurement of the rate of attachment of bacteriophage to cells. Bacteriophages: Methods and Protocols, Volume 1: Isolation, Characterization, and Interactions. USA: Humana press; 2009. 151–5 p. 10.1007/978-1-60327-164-6_15 19066819

[21] Wang Z, Zheng P, Ji W, Fu Q, Wang H, Yan Y, et al. SLPW: A virulent bacteriophage targeting methicillin-resistant Staphylococcus aureus in vitro and in vivo. Front Microbiol. 2016;7:934. doi: 10.3389/fmicb.2016.00934 27379064 PMC4908117

[22] Andrews S. FastQC: a quality control tool for high throughput sequence data. 2010. 2017.

[23] Seemann T. Shovill—Assemble Bacterial Isolate Genomes from Illumina Paired-End Reads. GitHub: San Francisco, CA, USA. 2020.

[24] Aziz RK, Bartels D, Best AA, DeJongh M, Disz T, Edwards RA, et al. The RAST Server: rapid annotations using subsystems technology. BMC genom. 2008;9(1):1–15. doi: 10.1186/1471-2164-9-75 18261238 PMC2265698

[25] NCBI. BLASTP [Available from: https://blast.ncbi.nlm.nih.gov/Blast.cgi?PROGRAM=blastp&PAGE_TYPE=BlastSearch&LINK_LOC=blasthome].

[26] Grant JR, Enns E, Marinier E, Mandal A, Herman EK, Chen C-y, et al. Proksee: in-depth characterization and visualization of bacterial genomes. Nucleic Acids Res. 2023:gkad326. doi: 10.1093/nar/gkad326 37140037 PMC10320063

[27] Lowe TM, Eddy SR. tRNAscan-SE: a program for improved detection of transfer RNA genes in genomic sequence. Nucleic Acids Res. 1997;25(5):955–64. doi: 10.1093/nar/25.5.955 9023104 PMC146525

[28] Tynecki P, Guziński A, Kazimierczak J, Jadczuk M, Dastych J, Onisko A. PhageAI-bacteriophage life cycle recognition with machine learning and natural language processing. BioRxiv. 2020:2020.07. 11.198606.

[29] Jia B, Raphenya AR, Alcock B, Waglechner N, Guo P, Tsang KK, et al. CARD 2017: expansion and model-centric curation of the comprehensive antibiotic resistance database. Nucleic Acids Res. 2016:gkw1004. doi: 10.1093/nar/gkw1004 27789705 PMC5210516

[30] Zankari E. Comparison of the web tools ARG-ANNOT and ResFinder for detection of resistance genes in bacteria. AAC. 2014;58(8):4986-. doi: 10.1128/AAC.02620-14 25028728 PMC4136053

[31] Liu B, Zheng D, Zhou S, Chen L, Yang J. VFDB 2022: a general classification scheme for bacterial virulence factors. Nucleic Acids Res. 2022;50(D1):D912–D7. doi: 10.1093/nar/gkab1107 34850947 PMC8728188

[32] Adriaenssens EM, Brister JR. How to name and classify your phage: an informal guide. Viruses. 2017;9(4):70. doi: 10.3390/v9040070 28368359 PMC5408676

[33] Sullivan MJ, Petty NK, Beatson SA. Easyfig: a genome comparison visualizer. Bioinformatics. 2011;27(7):1009–10. doi: 10.1093/bioinformatics/btr039 21278367 PMC3065679

[34] Moraru C, Varsani A, Kropinski AM. VIRIDIC—A novel tool to calculate the intergenomic similarities of prokaryote-infecting viruses. Viruses. 2020;12(11):1268. doi: 10.3390/v12111268 33172115 PMC7694805

[35] Meier-Kolthoff JP, Göker M. VICTOR: genome-based phylogeny and classification of prokaryotic viruses. Bioinformatics. 2017;33(21):3396–404. doi: 10.1093/bioinformatics/btx440 29036289 PMC5860169

[36] Ahmadi-Noorbakhsh S, Mirabzadeh Ardakani E, Sadighi J, Aldavood SJ, Farajli Abbasi M, Farzad-Mohajeri S, et al. Guideline for the care and use of laboratory animals in Iran. Lab animal. 2021;50(11):303–5. doi: 10.1038/s41684-021-00871-3 34621075

[37] Geng H, Zou W, Zhang M, Xu L, Liu F, Li X, et al. Evaluation of phage therapy in the treatment of Staphylococcus aureus-induced mastitis in mice. Folia Microbiol. 2020;65:339–51. doi: 10.1007/s12223-019-00729-9 31256341

[38] Zhu Y, Shang J, Peng C, Sun Y. Phage family classification under Caudoviricetes: A review of current tools using the latest ICTV classification framework. Front Microbiol. 2022;13:1032186. doi: 10.3389/fmicb.2022.1032186 36590402 PMC9800612

[39] NCBI. Taxonomy [Available from: https://www.ncbi.nlm.nih.gov/Taxonomy/Browser/wwwtax.cgi?id=542958].

[40] Hulo C, De Castro E, Masson P, Bougueleret L, Bairoch A, Xenarios I, et al. ViralZone: a knowledge resource to understand virus diversity. Nucleic Acids Res. 2011;39(suppl_1):D576-D82. doi: 10.1093/nar/gkq901 20947564 PMC3013774

[41] ViralZone. [Available from: https://viralzone.expasy.org/711].

[42] Guzinski A, Matusiak R. and Kazimierczak J. Staphylococcus phage 351Saur083PP, complete genome. 2023.

[43] Swift SM, Nelson DC. Complete genome sequence of Staphylococcus aureus phage GRCS. Genome Announc. 2014;2(2): doi: 10.1128/genomeA.00209-14 24723702 PMC3983291

[44] Sharifi F, Montaseri M, Yousefi MH, Shekarforoush SS, Berizi E, Wagemans J, et al. Isolation and characterization of two Staphylococcus aureus lytic bacteriophages “Huma” and “Simurgh”. Virol. 2024:110090. 10.1016/j.virol.2024.110090 38718447

[45] Campos B, Pickering AC, Rocha LS, Aguilar AP, Fabres-Klein MH, de Oliveira Mendes TA, et al. Diversity and pathogenesis of Staphylococcus aureus from bovine mastitis: Current understanding and future perspectives. BMC Vet Res. 2022;18(1):115. doi: 10.1186/s12917-022-03197-5 35331225 PMC8944054

[46] Khazaie F, Ahmadi E. Bovine subclinical mastitis-associated methicillin-resistant Staphylococcus aureus, selective genotyping and antimicrobial susceptibility profile of the isolates in Kurdistan province of Iran. Iran J Microbiol. 2021;13(1):65. doi: 10.18502/ijm.v13i1.5494 33889364 PMC8043834

[47] Ghaderi H, Mohammadzadeh A, Pajohi-alamoti M, Sadeghi-nasab A, Mahmoodi P, Goudarztalejerdi A. Molecular characterization and antibiotic resistance profile of methicillin-resistant Staphylococcus aureus (MRSA) strains isolated from milk samples of apparently healthy cattle in Hamedan, Iran. AJCMI. 2022;9(4):165–70.

[48] Selim A, Kelis K, AlKahtani MD, Albohairy FM, Attia KA. Prevalence, antimicrobial susceptibilities and risk factors of Methicillin resistant Staphylococcus aureus (MRSA) in dairy bovines. BMC Vet Res. 2022;18(1):293. doi: 10.1186/s12917-022-03389-z 35906609 PMC9336071

[49] Molineri AI, Camussone C, Zbrun MV, Archilla GS, Cristiani M, Neder V, et al. Antimicrobial resistance of Staphylococcus aureus isolated from bovine mastitis: Systematic review and meta-analysis. Prev Vet Med. 2021;188:105261. doi: 10.1016/j.prevetmed.2021.105261 33508662

[50] Zhang Z, Chen Y, Li X, Wang X, Li H. Detection of antibiotic resistance, virulence gene, and drug resistance gene of Staphylococcus aureus isolates from bovine mastitis. Microbiol Spectr. 2022;10(4):e00471–22. doi: 10.1128/spectrum.00471-22 35758746 PMC9431281

[51] Holtzman T, Globus R, Molshanski-Mor S, Ben-Shem A, Yosef I, Qimron U. A continuous evolution system for contracting the host range of bacteriophage T7. Sci Rep. 2020;10(1):307. doi: 10.1038/s41598-019-57221-0 31941920 PMC6962156

[52] Sofy AR, Abd El Haliem NF, Refaey EE, Hmed AA. Polyvalent phage CoNShP-3 as a natural antimicrobial agent showing lytic and antibiofilm activities against antibiotic-resistant coagulase-negative staphylococci strains. Foods. 2020;9(5):673. doi: 10.3390/foods9050673 32456227 PMC7278617

[53] Loc-Carrillo C, Abedon ST. Pros and cons of phage therapy. Bacteriophage. 2011;1(2):111–4. doi: 10.4161/bact.1.2.14590 22334867 PMC3278648

[54] Lin J, Du F, Long M, Li P. Limitations of phage therapy and corresponding optimization strategies: a review. Mol. 2022;27(6):1857. doi: 10.3390/molecules27061857 35335222 PMC8951143

[55] Hibstu Z, Belew H, Akelew Y, Mengist HM. Phage therapy: a different approach to fight bacterial infections. Biologics. 2022:173–86. doi: 10.2147/BTT.S381237 36225325 PMC9550173

[56] Pires DP, Costa AR, Pinto G, Meneses L, Azeredo J. Current challenges and future opportunities of phage therapy. FEMS Microbiol Rev. 2020;44(6):684–700. doi: 10.1093/femsre/fuaa017 32472938

[57] Rohde C, Resch G, Pirnay J-P, Blasdel BG, Debarbieux L, Gelman D, et al. Expert opinion on three phage therapy related topics: bacterial phage resistance, phage training and prophages in bacterial production strains. Viruses. 2018;10(4):178. doi: 10.3390/v10040178 29621199 PMC5923472

[58] Pradeep A, Ramasamy S, Veniemilda J, Kumar CV. Effect of ph & temperature variations on phage stability-a crucial prerequisite for successful phage therapy. Int J Pharm Sci Res. 2022;13:5178–82.

[59] Sinha S, Grewal RK, Roy S. Modeling bacteria–phage interactions and its implications for phage therapy. Adv Appl Microbiol. 103: Elsevier; 2018. p. 103–41. doi: 10.1016/bs.aambs.2018.01.005 29914656

[60] Abedon ST, Herschler TD, Stopar D. Bacteriophage latent-period evolution as a response to resource availability. Appl Environ Microbiol. 2001;67(9):4233–41. doi: 10.1128/AEM.67.9.4233-4241.2001 11526028 PMC93152

[61] Mohammadian F, Rahmani HK, Bidarian B, Khoramian B. Isolation and evaluation of the efficacy of bacteriophages against multidrug-resistant (MDR), methicillin-resistant (MRSA) and biofilm-producing strains of Staphylococcus aureus recovered from bovine mastitis. BMC Vet Res. 2022;18(1):406. doi: 10.1186/s12917-022-03501-3 36384653 PMC9670557

[62] Canchaya C, Fournous G, Chibani-Chennoufi S, Dillmann M-L, Brüssow H. Phage as agents of lateral gene transfer. Curr Opin Microbiol. 2003;6(4):417–24. doi: 10.1016/s1369-5274(03)00086-9 12941415

[63] Schneider CL. Bacteriophage-Mediated Horizontal Gene Transfer: Transduction. In: Harper DR, Abedon ST, Burrowes BH, McConville ML, editors. Bacteriophages: Biology, Technology, Therapy. Cham: Springer International Publishing; 2021. p. 151–92.

[64] Fernández L, Gutiérrez D, García P, Rodríguez A. The perfect bacteriophage for therapeutic applications—a quick guide. Antibiotics. 2019;8(3):126. doi: 10.3390/antibiotics8030126 31443585 PMC6783975

[65] Nicastro J, Wong S, Slavcev RA. Phage-Mediated Immunomodulation. Bacteriophage Applications ‐ Historical Perspective and Future Potential. Cham: Springer International Publishing; 2016. p. 69–82.

[66] Hodyra-Stefaniak K, Miernikiewicz P, Drapała J, Drab M, Jończyk-Matysiak E, Lecion D, et al. Mammalian Host-Versus-Phage immune response determines phage fate in vivo. Sci Rep. 2015;5(1):1–13. doi: 10.1038/srep14802 26440922 PMC4594097

[67] Berkson JD, Wate CE, Allen GB, Schubert AM, Dunbar KE, Coryell MP, et al. Phage-specific immunity impairs efficacy of bacteriophage targeting Vancomycin Resistant Enterococcus in a murine model. Nat Commun. 2024;15(1):2993. doi: 10.1038/s41467-024-47192-w 38582763 PMC10998888

[68] Iwano H, Inoue Y, Takasago T, Kobayashi H, Furusawa T, Taniguchi K, et al. Bacteriophage ΦSA012 has a broad host range against Staphylococcus aureus and effective lytic capacity in a mouse mastitis model. Biology. 2018;7(1):8. 29315249 10.3390/biology7010008 PMC5872034

[69] da Silva Duarte V, Dias RS, Kropinski AM, Campanaro S, Treu L, Siqueira C, et al. Genomic analysis and immune response in a murine mastitis model of vB_EcoM-UFV13, a potential biocontrol agent for use in dairy cows. Sci Rep. 2018;8(1):6845. doi: 10.1038/s41598-018-24896-w 29717158 PMC5931544

[70] Nale JY, McEwan NR. Bacteriophage therapy to control bovine mastitis: A review. Antibiotics. 2023;12(8):1307. doi: 10.3390/antibiotics12081307 37627727 PMC10451327

[71] Pedersen RR, Krömker V, Bjarnsholt T, Dahl-Pedersen K, Buhl R, Jørgensen E. Biofilm research in bovine mastitis. Front vet sci. 2021;8:656810. doi: 10.3389/fvets.2021.656810 34026893 PMC8138050

[72] Madsen JS, Burmølle M, Hansen LH, Sørensen SJ. The interconnection between biofilm formation and horizontal gene transfer. FEMS Immunol Med Microbiol. 2012;65(2):183–95. doi: 10.1111/j.1574-695X.2012.00960.x 22444301

[73] Gomes F, Saavedra MJ, Henriques M. Bovine mastitis disease/pathogenicity: evidence of the potential role of microbial biofilms. FEMS PD. 2016;74(3):ftw006. doi: 10.1093/femspd/ftw006 26772653

[74] Michaelis C, Grohmann E. Horizontal gene transfer of antibiotic resistance genes in biofilms. Antibiotics. 2023;12(2):328. doi: 10.3390/antibiotics12020328 36830238 PMC9952180

[75] Pires DP, Meneses L, Brandao AC, Azeredo J. An overview of the current state of phage therapy for the treatment of biofilm-related infections. Curr Opin Virol. 2022;53:101209. doi: 10.1016/j.coviro.2022.101209 35240424

