# Supplementary figures and images for: A novel broad-spectrum bacteriophage cocktail against methicillin-resistant Staphylococcus aureus: Isolation, characterization, and therapeutic potential in a mastitis mouse model
Source: PLoS One. 2025 Jan 15;20(1):e0316157. doi: 10.1371/journal.pone.0316157 (PMC11734958; doi:10.1371/journal.pone.0316157)

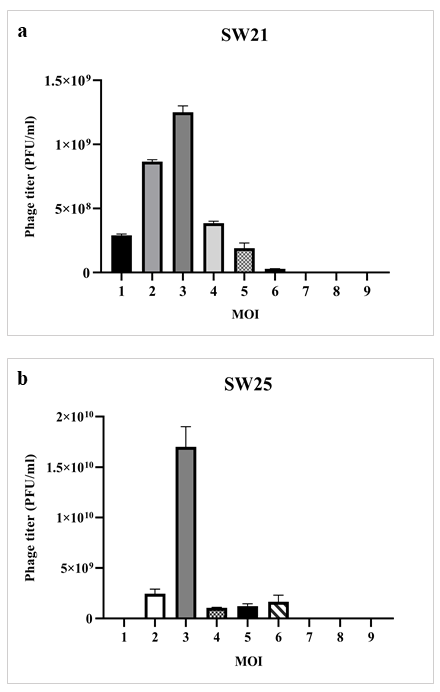

Supplement: S1 Fig — The optimal MOI of Staphylococcus phage vB_SauR_SW21 (a) and Staphylococcus phage vB_SauR_SW25 (b). Results are displayed as means ± SEM from duplicate experiments.1: MOI = 1000, 2: MOI = 100, 3: MOI = 10, 4: MOI = 1, 5: MOI = 0.1, 6: MOI = 0.01, 7: MOI = 0.001, 8: MOI = 0.0001, 9: MOI = 0.00001. (TIF) [file pone.0316157.s001.tif]

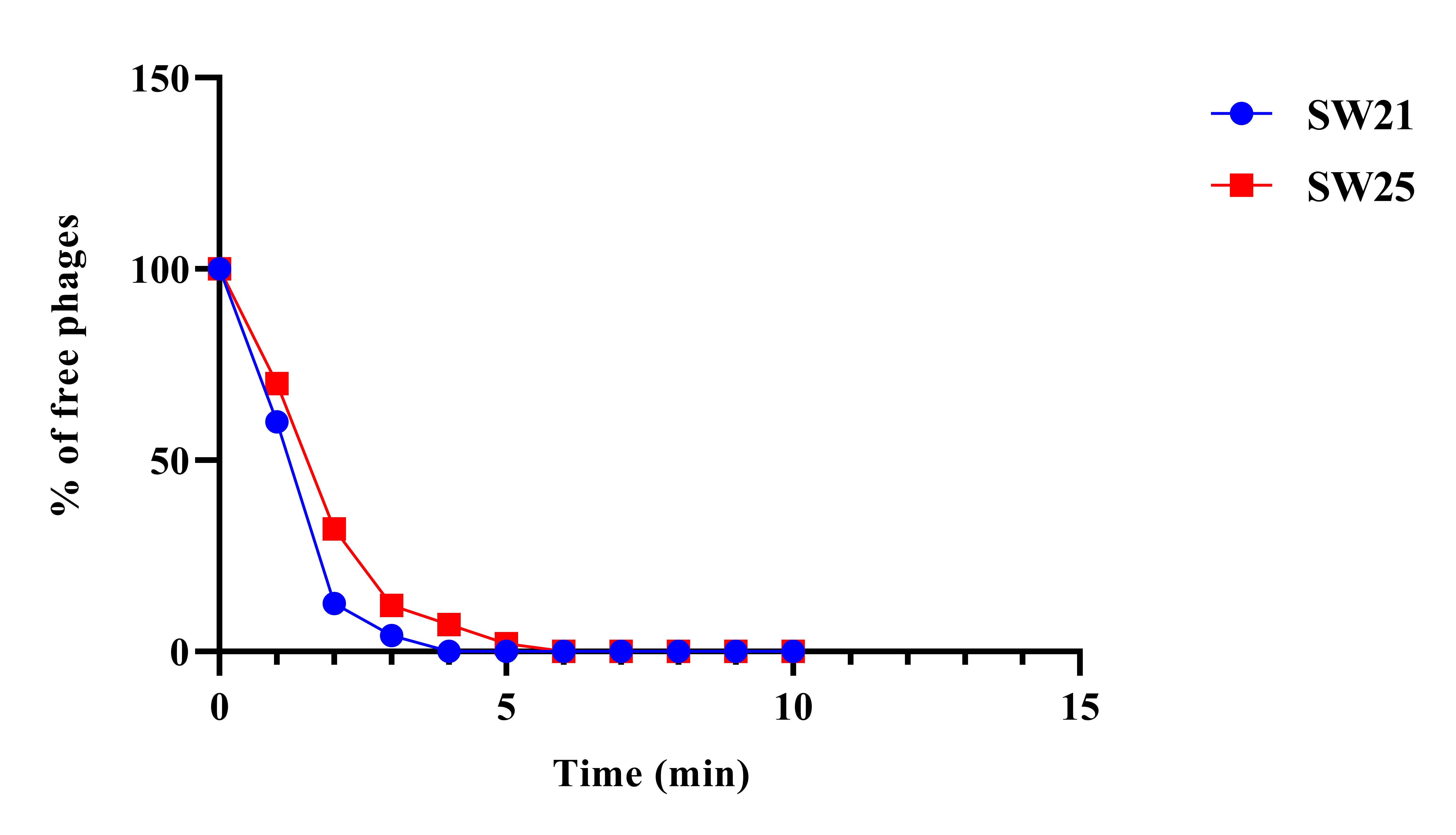

Supplement: S3 Fig — Adsorption rate of Staphylococcus phage vB_SauR_SW21 (a) and Staphylococcus phage vB_SauR_SW25 (b) to S. aureus ATCC 43300. The x-axis shows the incubation time (minutes) of phages with the host bacterium, while the y-axis displays the percentage of free (unadsorbed) phages. (TIF) [file pone.0316157.s003.tif]

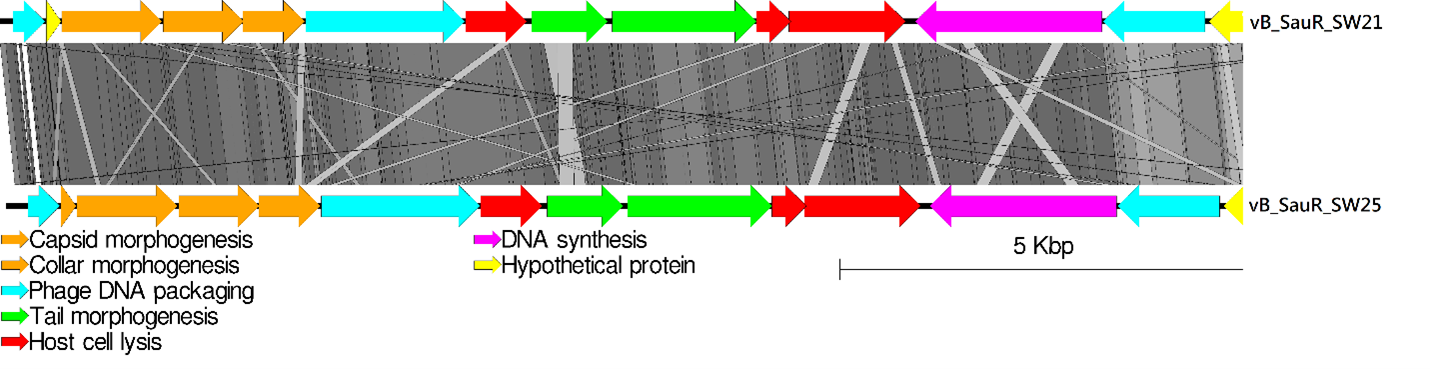

Supplement: S4 Fig — (TIF) [file pone.0316157.s004.tif]

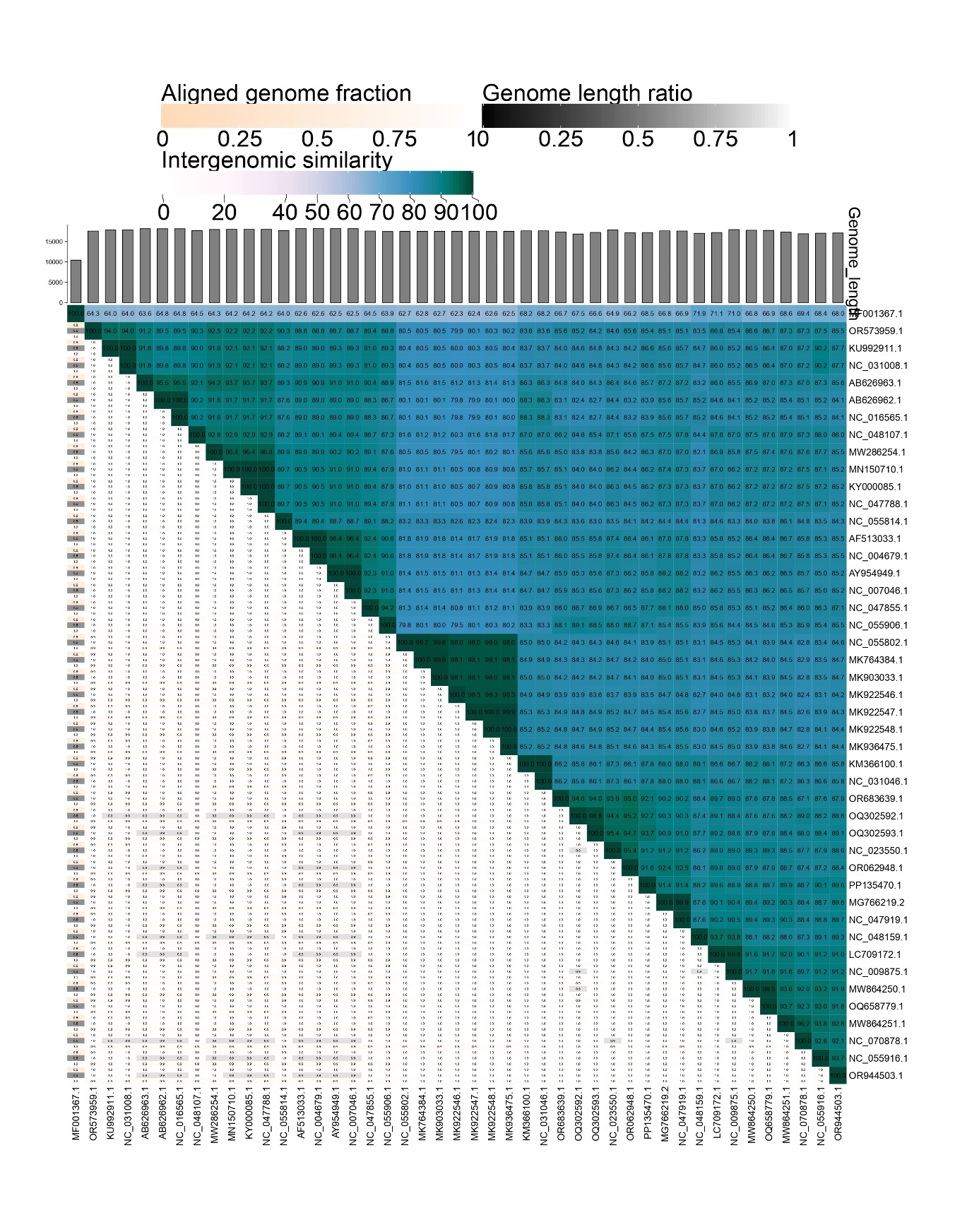

Supplement: S5 Fig — (DOCX) [file pone.0316157.s005.docx]

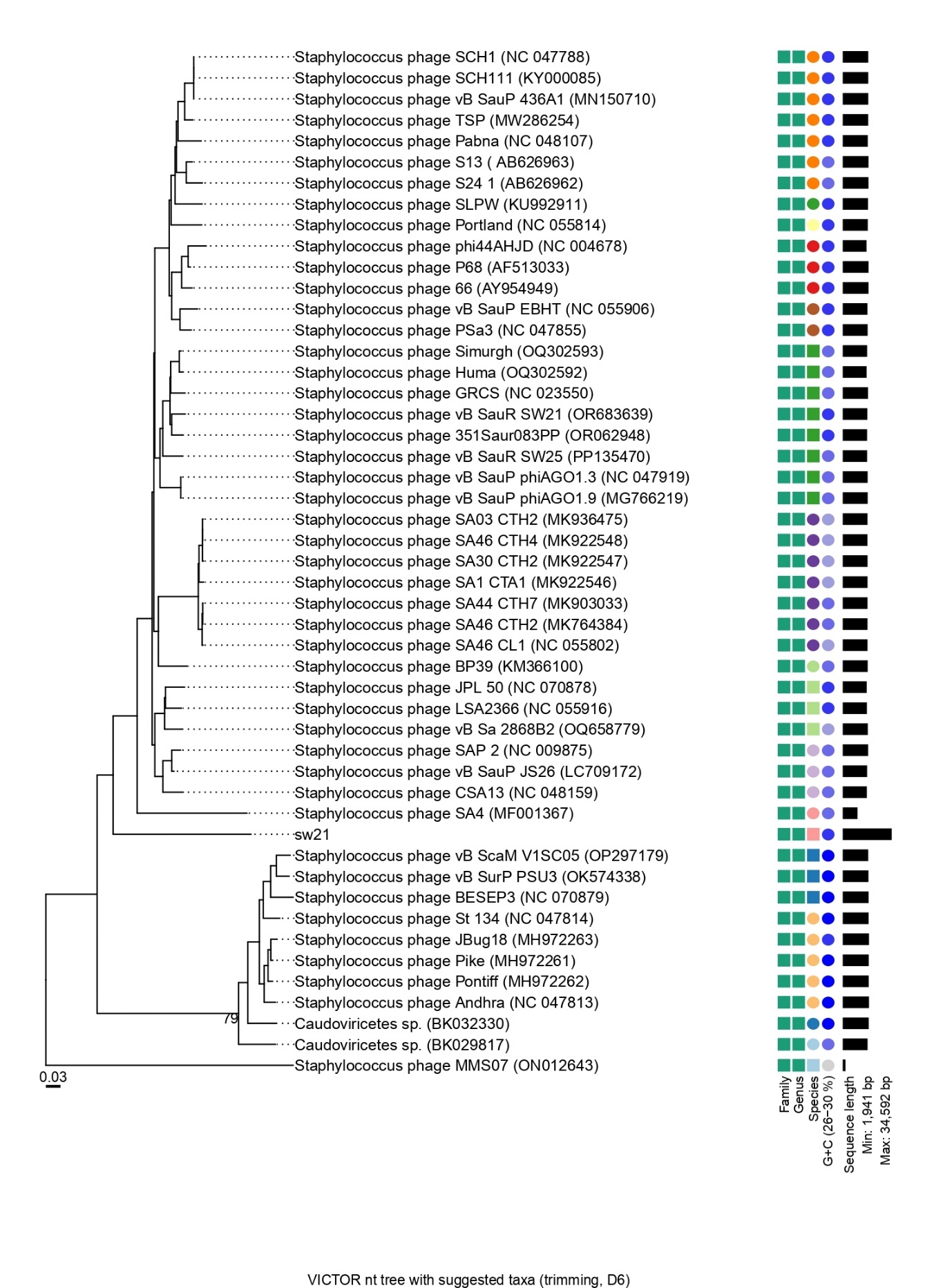

Supplement: S6 Fig — (DOCX) [file pone.0316157.s006.docx]
