# Supplementary material for: A novel broad-spectrum bacteriophage cocktail against methicillin-resistant Staphylococcus aureus: Isolation, characterization, and therapeutic potential in a mastitis mouse model
Source: PLoS One. 2025 Jan 15;20(1):e0316157. doi: 10.1371/journal.pone.0316157 (PMC11734958; doi:10.1371/journal.pone.0316157)
Supplement: S1 Table — (DOCX) [file pone.0316157.s007.docx]

| S1 Table. Genome annotation of Staphylococcus phage vB_SauR_SW21 | | | | | | | | |
| --- | --- | --- | --- | --- | --- | --- | --- | --- |
| ORF | Position (nt) | | strand | Codon | | Size (aa) | Predictive Function | Protein ID |
|  | From | To |  | Start | Stop |  |  |  |
| 1 | 163 | 555 | + | ATG | TAG | 130 | Chaperone protein DnaJ | [WPF65093.1](https://www.ncbi.nlm.nih.gov/protein/2621546119) |
| 2 | 568 | 750 | + | ATG | TAA | 60 | Hypothetical protein | WPF65094.1 |
| 3 | 757 | 1983 | + | ATG | TAA | 408 | Capsid and scaffold | [WPF65095.1](https://www.ncbi.nlm.nih.gov/protein/2621546121) |
| 4 | 1999 | 2982 | + | ATG | TAG | 327 | Phage collar | [WPF65096.1](https://www.ncbi.nlm.nih.gov/protein/2621546122) |
| 5 | 2975 | 3730 | + | ATG | TAA | 251 | Collar | WPF65097.1 |
| 6 | 3744 | 5687 | + | ATG | TAG | 647 | Phage major teichoic acid biosynthesis protein C (ACLAME 123) | WPF65098.1 |
| 7 | 5699 | 6448 | + | ATG | TAA | 249 | Phage lysin, N-acetylmuramoyl-L-alanine amidase (EC 3.5.1.28) | WPF65099.1 |
| 8 | 6511 | 7440 | + | ATG | TAA | 309 | Phage tail fibers | WPF65100.1 |
| 9 | 7496 | 9259 | + | ATG | TAA | 587 | Phage tail fibers | WPF65101.1 |
| 10 | 9261 | 9683 | + | ATG | TAA | 139 | Phage holin | WPF65102.1 |
| 11 | 9658 | 11091 | + | ATG | TAA | 477 | Hypothetical protein | WPF65103.1 |
| 12 | 13490 | 11205 | - | ATG | TAA | 761 | DNA polymerase (EC 2.7.7.7) | WPF65104.1 |
| 13 | 14752 | 13505 | - | ATG | TAA | 415 | Phage DNA packaging | WPF65105.1 |
| 14 | 15279 | 14800 | - | TTG | TAA | 159 | Hypothetical protein | WPF65106.1 |
| 15 | 15867 | 15445 | - | ATG | TAA | 140 | Hypothetical protein | WPF65107.1 |
| 16 | 16046 | 15870 | - | ATG | TAA | 58 | Hypothetical protein | WPF65108.1 |
| 17 | 16462 | 16094 | - | ATG | TAA | 122 | Single stranded DNA-binding protein, phage-associated | WPF65109.1 |
| 18 | 16722 | 16486 | - | ATG | TAA | 78 | Hypothetical protein | WPF65110.1 |
| 19 | 17042 | 16740 | - | ATG | TAG | 100 | Hypothetical protein | WPF65111.1 |

nt: nucleotide; aa: amino acid
