# Supplementary material for: A novel broad-spectrum bacteriophage cocktail against methicillin-resistant Staphylococcus aureus: Isolation, characterization, and therapeutic potential in a mastitis mouse model
Source: PLoS One. 2025 Jan 15;20(1):e0316157. doi: 10.1371/journal.pone.0316157 (PMC11734958; doi:10.1371/journal.pone.0316157)
Supplement: S2 Table — (DOCX) [file pone.0316157.s008.docx]

| S2 Table. Genome annotation of Staphylococcus phage vB_SauR_SW25 | | | | | | | | |
| --- | --- | --- | --- | --- | --- | --- | --- | --- |
| ORF | Position (nt) | | strand | Codon | | Size (aa) | Predictive Function | Protein ID |
|  | From | To |  | Start | Stop |  |  |  |
| 1 | 270 | 662 | + | ATG | TAG | 130 | Chaperone protein | [WVH10350.1](https://www.ncbi.nlm.nih.gov/protein/2664202473) |
| 2 | 676 | 858 | + | ATG | TAA | 60 | arstotzka protein | WVH10351.1 |
| 3 | 865 | 2100 | + | ATG | TAA | 411 | Capsid and scaffold protein | WVH10352.1 |
| 4 | 2116 | 3099 | + | ATG | TAG | 327 | Upper collar protein | WVH10353.1 |
| 5 | 3092 | 3847 | + | ATG | TAA | 251 | Lower Collar protein | WVH10354.1 |
| 6 | 3860 | 5803 | + | ATG | TAG | 647 | Phage major teichoic acid biosynthesis protein C (ACLAME 123) | WVH10355.1 |
| 7 | 5815 | 6564 | + | ATG | TAA | 249 | lysin, N-acetylmuramoyl-L-alanine amidase (EC 3.5.1.28) | WVH10356.1 |
| 8 | 6627 | 7556 | + | ATG | TAA | 309 | tail fiber protein | WVH10357.1 |
| 9 | 7613 | 9376 | + | ATG | TAA | 587 | tail fiber protein | WVH10358.1 |
| 10 | 9378 | 9800 | + | ATG | TAA | 140 | holin | WVH10359.1 |
| 11 | 9775 | 11199 | + | ATG | TAA | 474 | CHAP domain-containing protein | WVH10360.1 |
| 12 | 13597 | 11312 | - | ATG | TAA | 761 | DNA polymerase (EC 2.7.7.7) | WVH10361.1 |
| 13 | 14860 | 13613 | - | ATG | TAA | 415 | DNA packaging protein | WVH10362.1 |
| 14 | 15390 | 14908 | - | ATG | TAA | 160 | Hypothetical protein | WVH10363.1 |
| 15 | 15563 | 15393 | - | ATG | TAA | 56 | Hypothetical protein | WVH10364.1 |
| 16 | 15745 | 15566 | - | ATG | TAA | 59 | Hypothetical protein | WVH10365.1 |
| 17 | 16165 | 15797 | - | ATG | TAA | 122 | Single stranded DNA-binding protein | WVH10366.1 |
| 18 | 16425 | 16189 | - | ATG | TAA | 78 | Hypothetical protein | WVH10367.1 |
| 19 | 16745 | 16443 | - | ATG | TAG | 100 | Hypothetical protein | WVH10368.1 |

nt: nucleotide; aa: amino acid
