# Supplementary material for: A novel broad-spectrum bacteriophage cocktail against methicillin-resistant Staphylococcus aureus: Isolation, characterization, and therapeutic potential in a mastitis mouse model
Source: PLoS One. 2025 Jan 15;20(1):e0316157. doi: 10.1371/journal.pone.0316157 (PMC11734958; doi:10.1371/journal.pone.0316157)
Supplement: S3 Table — (DOCX) [file pone.0316157.s009.docx]

| **S3 Table. Characteristics of some closely-related Staphylococcus phages of the genus *Rosenblumvirus*** | | | | | | | | | | | | |
| --- | --- | --- | --- | --- | --- | --- | --- | --- | --- | --- | --- | --- |
| **Phage Name** | **Classification (species)** | **Geographic location** | **Genome size (bp)** | **G+C %** | **Number of CDS** | **Functional classification of phage proteins** | | | | | **GenBank Accession No.** | **Reference** |
|  |  |  |  |  |  | **Structural proteins, N (%)** | **Bacteriophage genome Packaging, N (%)** | **Phage DNA synthesis, N (%)** | **Membrane lysis and host cell wall degradation, N (%)** | **Hypothetical proteins, N (%)** |  |  |
| Staphylococcus phage vB_SauR_SW21 | Unclassified *Rosenblumvirus* | Tehran, Iran | 17369 | 29.5 | 19 | 7 (36.8) | 1 (5.3) | 2 (10.5) | 2 (10.5) | 7 (36.8) | OR683639.1 | This study |
| Phage 351Saur083PP | Unclassified *Rosenblumvirus* | Poland | 17209 | 29.3 | 19 | 8 (42.1) | 1 (5.3) | 1 (5.3) | 4 (21) | 5 (26.3) | OR062948.1 | (42) |
| Staphylococcus aureus Phage GRCS | *Rosenblumvirus* GRCS | USA | 17869 | 28.9 | 21 | 6 (28.6) | 1 (4.8) | 1 (4.8) | 3 (14.2) | 10 (47.6) | NC_023550 | (43) |
| Staphylococcus phage Huma | Unclassified *Rosenblumvirus* | Shiraz, Iran | 16853 | 29.3 | 19 | 6 (31.6) | 1 (5.3) | 2 (10.5) | 3 (15.8) | 7 (36.8) | OQ302592.1 | (44) |
| Staphylococcus phage Simorgh | Unclassified *Rosenblumvirus* | Shiraz, Iran | 17245 | 29 | 19 | 6 (31.6) | 1 (5.3) | 2 (10.5) | 2 (10.5) | 8 (42.1) | OQ302593.1 | (44) |
| Staphylococcus phage vB_SauR_SW25 | Unclassified *Rosenblumvirus* | Tehran, Iran | 17223 | 29.24 | 19 | 7 (36.8) | 1 (5.3) | 2 (10.5) | 4 (21) | 5 (26.3) | PP135470.1 | This study |
