# Supplementary material for: A novel broad-spectrum bacteriophage cocktail against methicillin-resistant Staphylococcus aureus: Isolation, characterization, and therapeutic potential in a mastitis mouse model
Source: PLoS One. 2025 Jan 15;20(1):e0316157. doi: 10.1371/journal.pone.0316157 (PMC11734958; doi:10.1371/journal.pone.0316157)
Supplement: S4 Table — (DOCX) [file pone.0316157.s010.docx]

| **S4 Table. Blood parameters in different mice groups after 24 and 48 hours.** | | | | | | | | | | | | | | |
| --- | --- | --- | --- | --- | --- | --- | --- | --- | --- | --- | --- | --- | --- | --- |
|  |  | **C.B.C** | | | | | | | | **Platelet** | | | | |
| **Groups** | **Time** | RBC (ml/μl) | Hemoglobin (g/dl) | Hematocrit (%) | M.C.V* (fl) | M.C.H* (pg) | M.C.H.C* (g/dl) | R.D.W-CV* (%) | R.D.W-SD* (fl) | Platelet Count (1000/µl) | P.D.W* (fl) | M.P.V* (fl) | P-LCR* (%) | P.C.T* (%) |
| Phage therapy 1 | 24 h | 6.415 | 10.55 | 38.85 | 69.35 | 17.9 | 26.6 | 22.2 | 47.6 | 725 | 6.75 | 6.8 | 5.65 | 0.48 |
|  | 48 h | 9.385 | 13.9 | 44.4 | 47.3 | 14.8 | 31.3 | 21.5 | 31.05 | 970 | 6.4 | 6.05 | 3.25 | 0.59 |
| Phage therapy 2 | 24 h | 8.78 | 12 | 40.75 | 46.4 | 13.7 | 29.45 | 20.3 | 28.95 | 1121 | 6.5 | 6.3 | 3.1 | 0.705 |
|  | 48 h | 9.13 | 13.6 | 44.9 | 49.2 | 14.85 | 30.3 | 21.75 | 33.55 | 1064 | 6.5 | 6.4 | 4.15 | 0.675 |
| Phage control | 24 h | 10.255 | 14.65 | 49.3 | 48.05 | 14.3 | 29.75 | 22.65 | 33.15 | 959 | 7.35 | 6.55 | 4.75 | 0.63 |
|  | 48 h | 8.98 | 13 | 43.55 | 48.3 | 14.45 | 30 | 22.7 | 35.35 | 885 | 7.65 | 6.95 | 6.6 | 0.565 |
| Positive control | 24 h | 8.14 | 10.65 | 35.45 | 43.55 | 13.05 | 30.05 | 26.8 | 17.7 | 961 | 3.25 | 3.15 | 1.75 | 0.395 |
|  | 48 h | 9.255 | 13.5 | 45.85 | 49.55 | 14.6 | 29.45 | 22.4 | 35.05 | 829 | 6.55 | 6.2 | 3.25 | 0.515 |
| Negative control | 24 h | 8.65 | 12.85 | 42.45 | 49.1 | 14.85 | 30.25 | 20.7 | 32.3 | 1031 | 6.45 | 6.4 | 3.75 | 0.655 |
|  | 48 h | 9.935 | 13.8 | 45.65 | 46 | 13.9 | 30.2 | 24.75 | 37 | 837 | 7.15 | 6.4 | 4.1 | 0.54 |
| Blank control | 24 h | 9.3 | 13.4 | 43.35 | 46.65 | 14.4 | 30.85 | 24.2 | 35.65 | 901 | 7.05 | 6.35 | 3.75 | 0.58 |
|  | 48 h | 9.87 | 14.7 | 46.55 | 47.3 | 14.95 | 31.55 | 23.15 | 34.25 | 906 | 6.95 | 6.25 | 2.8 | 0.57 |

* M.C.V: Mean corpuscular volume, M.C.H: Mean corpuscular hemoglobin, M.C.H.C: Mean corpuscular hemoglobin concentration, R.D.W-CV: Red Cell Distribution Width (RDW)- coefficient of variation (CV), R.D.W-SD: Red Cell Distribution Width (RDW)- standard deviation (SD), P.D.W: Platelet Distribution Width, M.P.V: Mean Platelet Volume, P-LCR: Platelet larger cell ratio, P.C.T: Plateletcrit.
